# Na_v_1.2 channel mutations preventing fast inactivation lead to *SCN2A* encephalopathy

**DOI:** 10.1093/brain/awae213

**Published:** 2024-06-28

**Authors:** Géza Berecki, Elaine Tao, Katherine B Howell, Rohini K Coorg, Erik Andersen, Kris Kahlig, Markus Wolff, Ben Corry, Steven Petrou

**Affiliations:** Ion Channels and Human Disease Group, The Florey Institute of Neuroscience and Mental Health, University of Melbourne, Parkville, VIC 3052, Australia; Department of the Florey Institute, University of Melbourne, Parkville, VIC 3050, Australia; Division of Biomedical Science and Biochemistry, Research School of Biology, Australian National University, Canberra, ACT 2601, Australia; Department of Neurology, Royal Children’s Hospital, Parkville, VIC 3052, Australia; Neuroscience, Murdoch Children’s Research Institute, Parkville, VIC 3052, Australia; Division of Neurology and Developmental Neuroscience, Department of Pediatrics, Texas Children’s Hospital, Baylor College of Medicine, Houston, TX 77030, USA; Department of Paediatrics and Child Health, University of Otago, Wellington 6242, New Zealand; Praxis Precision Medicines, Inc., Cambridge, MA 02142, USA; Swiss Epilepsy Center, Klinik Lengg, Zürich 8001, Switzerland; Division of Biomedical Science and Biochemistry, Research School of Biology, Australian National University, Canberra, ACT 2601, Australia; Ion Channels and Human Disease Group, The Florey Institute of Neuroscience and Mental Health, University of Melbourne, Parkville, VIC 3052, Australia; Department of the Florey Institute, University of Melbourne, Parkville, VIC 3050, Australia; Praxis Precision Medicines, Inc., Cambridge, MA 02142, USA

**Keywords:** *SCN2A* gene, Na_v_1.2 channel, fast inactivation, dynamic action potential clamp, epilepsy, developmental and epileptic encephalopathy

## Abstract

*SCN2A* gene-related early-infantile developmental and epileptic encephalopathy (EI-DEE) is a rare and severe disorder that manifests in early infancy. *SCN2A* mutations affecting the fast inactivation gating mechanism can result in altered voltage dependence and incomplete inactivation of the encoded neuronal Na_v_1.2 channel and lead to abnormal neuronal excitability.

In this study, we evaluated clinical data of seven missense Na_v_1.2 variants associated with DEE and performed molecular dynamics simulations, patch-clamp electrophysiology and dynamic clamp real-time neuronal modelling to elucidate the molecular and neuron-scale phenotypic consequences of the mutations.

The N1662D mutation almost completely prevented fast inactivation without affecting activation. The comparison of wild-type and N1662D channel structures suggested that the ambifunctional hydrogen bond formation between residues N1662 and Q1494 is essential for fast inactivation. Fast inactivation could also be prevented with engineered Q1494A or Q1494L Na_v_1.2 channel variants, whereas Q1494E or Q149K variants resulted in incomplete inactivation and persistent current. Molecular dynamics simulations revealed a reduced affinity of the hydrophobic IFM-motif to its receptor site with N1662D and Q1494L variants relative to wild-type. These results demonstrate that the interactions between N1662 and Q1494 underpin the stability and the orientation of the inactivation gate and are essential for the development of fast inactivation. Six DEE-associated Na_v_1.2 variants, with mutations mapped to channel segments known to be implicated in fast inactivation were also evaluated. Remarkably, the L1657P variant also prevented fast inactivation and produced biophysical characteristics that were similar to those of N1662D, whereas the M1501V, M1501T, F1651C, P1658S and A1659V variants resulted in biophysical properties that were consistent with gain-of-function and enhanced action potential firing of hybrid neurons in dynamic action potential clamp experiments. Paradoxically, low densities of N1662D or L1657P currents potentiated action potential firing, whereas increased densities resulted in sustained depolarization.

Our results provide novel structural insights into the molecular mechanism of Na_v_1.2 channel fast inactivation and inform treatment strategies for *SCN2A*-related EI-DEE. The contribution of non-inactivating Na_v_1.2 channels to neuronal excitability may constitute a distinct cellular mechanism in the pathogenesis of *SCN2A*-related DEE.

## Teaser

In a functional-clinical study of *SCN2A* variants, Berecki *et al.* provide new insights into the molecular mechanism of Na_v_1.2 channel inactivation, predict the impact of non-inactivating Na_v_1.2 variants on neuronal function and inform treatment strategies for *SCN2A-*related developmental and epileptic encephalopathy.

## Introduction

The *SCN2A* gene-encoded voltage-gated Na_v_1.2 channels are predominantly expressed in the excitatory neurons of the CNS, where they contribute to the generation and propagation of action potentials. *De novo* missense mutations in *SCN2A* can lead to neurodevelopmental disorders of various severity, including early-infantile developmental and epileptic encephalopathy (DEE), a rare severe disorder caused by Na_v_1.2 gain-of-function.^1-3^

Na_v_1.2 channels activate in response to depolarizing stimuli to allow the influx of Na^+^ ions. Activation is followed by fast inactivation, a mechanism which terminates the Na^+^ conductance even in the continued presence of depolarization. This mechanism is important for controlling neuronal excitability by preventing rapid refiring and allowing the membrane potential to return to resting values once the signal has been sent. Fast inactivation of sodium channels has been intensely studied for nearly 80 years.^4-10^ Channel activation is initiated by the rapid movement of segment 4 (S4) voltage sensors in domains I-III (S4_DI-III_), leading to pore opening followed by the slower movement of S4_DIV,_^11^ which represents the rate-limiting step for the development of fast inactivation and the recovery from this state.^12^ A sequence of three conserved hydrophobic amino acids (IFM motif) in the DIII-IV linker (inactivation gate) is essential for fast inactivation since mutations of these residues are able to slow or completely remove fast inactivation.^13^ In rat brain sodium channels, alanine scanning mutagenesis of the S4-5_DIV_ region and subsequent two-microelectrode voltage clamp experiments showed incomplete fast inactivation with F1651A or L1660A channels and a nearly abolished fast inactivation with the N1662A mutant relative to wild-type.^14^ It has been suggested that the mutated residues form part of the inactivation gate’s IFM motif receptor.^6,14^

The availability of sodium channel structures provides a unique opportunity to further unravel the fast inactivation mechanism at the molecular level.^15-19^ This process involves a series of electrostatic interactions between amino acid residues located on S4_DIV_, S4-5_DIV_ linker, DIII-IV linker (inactivation gate), S6_DIII_ and S6_DIV_ of the intracellular pore module, and the C-terminal domain (CTD).^15^ A hydrophobic receptor site mapped to residues in the S4-5_DIII_ and S4-5_DIV_ linkers and outside of the helices lining the activation gate represents the IFM motif binding site.^15,18,20^ Recent data suggest that in addition to IFM motif binding, the closure of two hydrophobic rings at the bottom of S6 helices is also needed to complete fast inactivation.^21^ However, despite the experimental data and the novel cryo-electron microscopy (EM) structures of various neuronal Na_v_ channels,^16,17,22,23^ fundamental questions regarding the precise molecular interactions mediating Na_v_1.2 fast inactivation still prevail.

Early-infantile DEE-related Na_v_1.2 channel variants often show impaired biophysical properties due to a changed voltage dependence, time course and/or extent of fast inactivation. Patients are at high risk of premature mortality from direct and indirect effects of seizures, which typically begin within days of birth and are often difficult to control with antiseizure medications. Understanding the mechanisms of ion channel dysfunction leading to abnormal neuronal excitability should facilitate the interpretation of the neurological disease and the implementation of mechanism-targeted therapies.

In this study, we assessed the biophysical and structural consequences of the N1662D missense mutation identified in a patient with severe early-infantile DEE. This mutation is predicted to cause local structural changes that severely alter fast inactivation and neuronal excitability. We used engineered Na_v_1.2 channel variants to probe the role of hydrogen bond formation between residues N1662 (located in the cytoplasmic end of S5_DIV_) and Q1494 (located in the DIII-IV linker) in fast inactivation. Similarly, we also assessed six DEE-associated Na_v_1.2 missense mutations located nearby the N1662 residue (L1657P, P1658S, A1659V), the S4-5_DIV_ linker (F1651C) or the DIII-IV linker (M1501V, M1501T) to determine their impact on fast inactivation and neuronal excitability. To understand the structural changes caused by the N1662D mutation relative to wild-type, we used molecular dynamics (MD) simulations and evaluated the binding stability of the IFM motif and other key interactions between residues in the S4-S5_DIV_ linker and DIII-IV linker. The results suggest that the N1662-Q1494 interaction plays a critical role in maintaining a stably bound inactivation gate, and mutations of either residue lead to disrupted fast inactivation.

## Materials and methods

### SCN2A variants and patient data

Pathogenic *SCN2A* variants were identified through the *SCN2A* International Natural History Study (NHS) (c.4984A>G, p.N1662D); ClinVar database (https://www.ncbi.nlm.nih.gov/clinvar/) (c.4502T>C, p.M1501T; c.4972C>T, p.P1658S); Simons Searchlight database (SSDb) (https://www.sfari.org/resource/simons-searchlight/) (c.4501A>G, p.M1501V); or referred through a network of collaborating clinicians (c.4970T>, p.L1657P). The c.4952T>G, p.F1651C and c.4976C>T, p.A1659V variants have been reported previously.^24,25^ The study was approved by the Human Research Ethics Committees of the Royal Children’s Hospital and Austin Health Melbourne, State Medical Association of Berlin and Simons Foundation. Written informed consent was obtained for the individuals with the L1657P and N1662D variants, whose clinical data are presented here.

### Na_v_1.2 channel mutagenesis

For all variants, the adult *SCN2A* isoform^26^ was used as a template. The N1662D, Q1494A, Q1494E, Q1494L and Q1494K variants were synthesized using a QuikChange mutagenesis kit (Agilent Technologies) with custom-made forward (F) and reverse (R) primers (Bioneer Pacific) detailed in Supplementary Table 1. All sequences were verified and confirmed at the Australian Genome Research Facility (Melbourne, Victoria, Australia). The F1651C, M1501V, M1501T, L1657P, P1658S and A1659V variants were custom-made (Genscrip).

### Cell culture and transfection

Chinese hamster ovary (CHO) cells were cultured and transiently co-transfected with wild-type or mutant Na_v_1.2 channel variant (5 μg) and enhanced green fluorescent protein (1 μg) using Lipofectamine 3000 kit (Thermo Fisher Scientific), as previously described.^27^ Cells were incubated at 37°C in 5% CO_2_ for 24 h. Three to four days post-transfection, the cells were dissociated using TrypLE Express (Thermo Fisher Scientific Australia Pty Ltd) and plated on glass coverslips for electrophysiological recordings.

### Electrophysiology data and analysis

Sodium currents through Na_v_1.2 channel variants (I_Na_) were recorded using the whole-cell configuration of the patch-clamp technique using an Axopatch 200B amplifier (Molecular Devices) as previously described.^27,28^ The CHO cells were superfused with extracellular solution containing 145 mM NaCl, 5 mM CsCl, 2 mM CaCl_2_, 1 mM MgCl_2_, 5 mM glucose, 5 mM sucrose and 10 mM HEPES (pH = 7.4 with NaOH), at a rate of ∼0.2 ml/min. Patch pipettes of ∼1.5 MΩ resistance were pulled from borosilicate glass capillaries (GC150TF-7.5, Harvard Apparatus Ltd.) and filled with intracellular (pipette) solution containing 5 mM CsCl, 120 mM CsF, 10 mM NaCl, 11 mM EGTA, 1 mM CaCl_2_, 1 mM MgCl_2_, 2 mM Na_2_ATP and 10 mM HEPES (pH = 7.3 with CsOH). Current and potentials were low-pass filtered (cut-off frequency 10 kHz) and digitized at 50 kHz. Series resistance was compensated by ≥85%, and potentials were corrected for the estimated liquid junction potential. The leak and capacitive currents were corrected using a −P/4 pulse protocol, except when using steady-state inactivation and recovery from fast inactivation protocols. The voltage protocols assessing I_Na_ activation, steady-state inactivation, recovery from fast inactivation, persistent I_Na_ (I_Na-P_), and I_Na_ kinetics were described previously^28^ and are shown as insets in the figures.

Dynamic action potential clamp (DAPC) recordings were performed by implementing scaled wild-type or A1329D I_Na_ in a biophysically realistic axon initial segment (AIS) model, as previously described.^28^ Unless specified otherwise, the virtual sodium conductance of the AIS model was set to zero, whereas the virtual K_v_ channel and the virtual Na_v_1.6 channel conductance values were set to gK_v_ = 2 (twice the original gK_v_) and gNa_v_1.6 = 0.4, respectively.^28^ Action potential firing was elicited using step current injections of 1 s duration in 2-pA increments between −2 and +24 pA. In all DAPC experiments, the membrane potential (V_m_), stimulus current, *in silico* I_Kv_, *in silico* I_Nav1.6_ and I_Nav1.2_ were simultaneously recorded.

Current densities were determined by dividing the inward peak I_Na_ current amplitudes by cell capacitance (C_m_). The voltage-dependence of the activation was determined from a holding membrane potential (HP) of −120 mV, using depolarizing voltage steps of 40 ms duration in 5 mV increments in the voltage range between −80 and +55 mV. Current density values were plotted against membrane voltage to obtain current density–voltage relationships. Conductance (G) was determined according to the equation G = I/(V−V_rev_), where V_rev_ is the reversal potential for Na^+^. Normalized conductance values (G/Gmax) were plotted against membrane potential to obtain activation curves. The voltage dependence of steady-state fast inactivation was determined from a HP of −120 mV using preconditioning voltage steps of 100 ms duration in 5 mV increments in the voltage range between −80 and +20 mV, followed by a 20-ms test pulse to −10 mV to test the availability of the sodium current. Activation and inactivation curves were fit using the Boltzmann equation as follows:


(1) $$\frac{G}{G_{\max}}=\frac{1}{\left[1+e^{(V-V_{0.5})/k}\right]}$$

where V is the membrane voltage, V_0.5_ is the voltage for half-maximal activation or inactivation (V_0.5, act_ or V_0.5, inact_, respectively) and k is the slope factor. I_Na-P_ was determined after −P/4 leak correction, 40 ms after the onset of a depolarizing voltage step and was expressed as percent of peak I_Na_.^28^ I_Na-P_ was not determined for the engineered Na_v_1.2 variants (Q1494A, Q1494E, Q1494L, and Q1494K) and for the two pathogenic Na_v_1.2 variants (N1662D and L1657P). Q1494A, Q1494L, N1662D and L1657P exhibited non-inactivating inward current amplitudes ≥50% of the inward peak 40 ms after the onset of a depolarizing voltage step.

The time constant of the recovery from fast inactivation was determined by measuring current amplitudes during paired-pulse (P1 and P2) depolarizations^28^ and fitting a single exponential equation to data, as follows:


(2) $$\frac{I}{I_{\max}}=1-e^{-t/\tau }$$

where I_max_ is the current amplitude elicited by P1, I is the current amplitude elicited by P2, and t is the time between P1 and P2. For variants exhibiting a relatively large fraction of non-inactivating current (Q1494A, Q1494E, Q1494L, N1662D and L1657P), the inactivated current fraction was first determined as the difference between the inward peak current amplitudes during P1 and P2 when using t = 0.1 ms. The recovery of the inactivated current fraction was plotted against t and a single exponential equation was fitted to the data as described above.

The firing frequency of the variants relative to wild-type was determined using Clampfit 10.7 software (Molecular Devices). The mean firing frequency was plotted against the depolarizing stimulus current amplitude resulting in input-output relationships.

### 3D structural modelling

The voltage-gated Na_v_1.2 channel structure was downloaded from the Protein Data Bank^29^ (PDB accession no. 6J8E).^16^ The *in silico* mutations of Na_v_1.2 were introduced using Pymol 2.3.2 (Schroedinger LLC).

### Molecular dynamics simulations

Systems were set up for wild-type, N1662D, Q1494L, L1657P, M1501V and F1651C. The proteins were embedded within a pure 1-palmytoyl-2-oleoyl-sn-glycero-3-phosphatidylcholine (POPC) bilayer and solvated with 0.15 M NaCl solution using CHARMM-GUI.^30^ Mutations were introduced accordingly for each of the relevant systems. Systems were 140 Å along the plane of the membrane (*x* and *y* directions) and 120 Å in the *z* direction.

Simulations were conducted using Amber20,^31,32^ using the following forcefields: ffSB19 protein,^33^ Lipid22,^34^ OPC water^35^ and 12–6 ion parameters.^36^ All systems were equilibrated via minimizing, heating, pressurizing, a short 50 ns of protein backbone restrained simulation (at 5 kJ/mol), and gradual reduction of restraint over 24 ns. Each system was then simulated for five replicates of 1 µs each at 1 bar and 310 K, using the Monte Carlo barostat^37^ and Langevin thermostat,^38^ respectively. Hydrogen mass repartitioning^39^ was used to increase the timestep to 4 fs and hydrogen bonds were constrained via the SHAKE algorithm.^40^ Periodic boundary conditions and a 10 Å van der Waals cut-off were used. RMSD (root mean square deviation), RMSF (root mean square fluctuation) and distance calculations were performed using MDAnalysis.^41^ Hydrogen bond interactions were assessed using ProLIF.^42^ Representative snapshots from MD trajectories were produced in Visual Molecular Dynamics (VMD) software.^43^

Replica exchange with solute tempering (REST2) simulations were set up for wild-type, N1662D and Q1494L using the CHARMM-GUI Enhanced Sampler.^44^ Hamiltonians in the DIII-IV linker (residues 1486–1518) and side-chain atoms in the linker binding residues (1321–1330, 1476, 1479–1480, 1483, 1651–1663, 1769–1777) were scaled. Sixteen replicas were used at 310.15, 326.37, 343.20, 360.67, 378.75, 397.62, 417.08, 437.27, 458.17, 479.88, 502.42, 525.80, 550.06, 575.24, 601.42, 620.30 K respectively. Systems were equilibrated for 10 ns without scaled Hamiltonians; then each replica was equilibrated for 10 ns with scaled Hamiltonians. REST2 was run for 500 ns, with a time step of 2 fs and attempting exchange every 1000 steps. Simulations were conducted with GROMACS 2022^45^ using the CHARMM36m^46^ forcefield and TIP3P water,^47^ with the pressure set at 1 bar using the Parinello-Rahman barostat^48^ and the global temperature set to 310 K using the Noose-Hoover thermostat.^49^ Analysis was conducted on the lowest temperature replica.

### Statistical information

Data are presented as mean ± standard error of the mean. Electrophysiological data were analysed in Clampfit 9.2 (Molecular Devices). Statistical analyses were carried out using GraphPad Prism 9.0 (La Jolla, CA, USA) and Origin 2023 (Microcal Software Inc., Northampton, MA). One-way ANOVA followed by Dunnett’s *post hoc* test was used to evaluate the properties of Na_v_1.2 channel variants relative to wild-type. Pearson’s correlation analysis was used to determine the strength of linear association between the fraction of N1662D I_Na_ and the V_m_ value showing sustained depolarization in DAPC experiments, and the correlation coefficient, *r*. Differences were considered statistically significant if *P* < 0.05; *n*, the number of individual experiments, is included in the figures and Table 1.

**Table 1:** Biophysical characteristics of the engineered and pathogenic Na_v_1.2 variants relative to wild-type

Variant	I_Na_ density at −10 mV (pA/pF)	V_0.5,act_ (mV)	V_0.5,inact_ (mV)	I_Na-P_ at −10 mV (% of peak)	t recovery (ms)
WT	458.3 ± 55	−17.56 ± 0.42	−51.70 ± 0.96	1.06 ± 0.29	1.19 ± 0.03
*n*	22	22	22	22	18
*P*-value	–	–	–	–	–
N1662D	95.2 ± 17****	−17.24 ± 0.44^NS^	−39.56 ± 1.3****	ND	0.54 ± 0.02****
*n*	16	16	16	–	14
*P*-value	<0.0001	0.999	<0.0001	–	<0.0001
Q1494A	212.7 ± 28**	−13.39 ± 0.47****	−36.75 ± 2.1****	ND	0.51 ± 0.04****
*n*	6	6	6	–	5
*P*-value	0.048	<0.0001	<0.0001	–	<0.0001
Q1494E	195.8 ± 54**	−16.01 ± 0.72^NS^	−36.15 ± 1.3****	ND	0.61 ± 0.04****
*n*	6	6	6	–	6
*P*-value	0.002	0.463	<0.0001	–	<0.0001
Q1494L	103.0 ± 14****	−15.28 ± 0.74*	−43.02 ± 1.4****	ND	0.69 ± 0.03****
*n*	7	7	7	–	7
*P*-value	<0.0001	0.044	<0.0001	−	<0.0001
Q1494K	38.3 ± 7.9****	−9.03 ± 0.58****	−43.22 ± 0.46****	ND	1.01 ± 0.08**
*n*	9	9	8	–	8
*P*-value	<0.0001	<0.0001	<0.0001	–	0.0038
F1651C	461.5 ± 60^NS^	−16.12 ± 0.59^NS^	−38.92 ± 0.53****	4.86 ± 0.57****	0.67 ± 0.02****
*n*	7	7	7	7	6
*P*-value	>0.999	0.482	<0.0001	<0.0001	<0.0001
M1501V	265.4 ± 51*	−10.81 ± 0.61****	−42.63 ± 0.68****	4.04 ± 0.79**	0.84 ± 0.04****
*n*	7	7	6	7	7
*P*-value	0.032	<0.0001	0.0001	0.0028	<0.0001
M1501T	275.2 ± 27^NS^	−18.18 ± 0.58^NS^	−46.37 ± 0.54^NS^	3.63 ± 0.96*	0.69 ± 0.01****
*n*	6	6	6	6	6
*P*-value	0.076	0.996	0.007	0.021	<0.0001
L1657P	109.8 ± 24****	−16.51 ± 0.84^NS^	−44.82 ± 3.7**	ND	0.62 ± 0.03****
*n*	7	7	7	–	6
*P*-value	<0.0001	0.844	0.0036	–	<0.0001
P1658S	140.6 ± 22***	−14.34 ± 0.57***	−42.62 ± 0.68****	5.91 ± 0.99****	0.59 ± 0.02****
*n*	9	9	9	9	7
*P*-value	<0.0001	0.0002	<0.0001	<0.0001	<0.0001
A1659V	361.3 ± 50^NS^	−17.00 ± 0.63^NS^	−46.66 ± 0.60*	5.64 ± 0.43****	0.77 ± 0.05****
*n*	10	10	10	10	9
*P*-value	0.562	0.992	0.026	<0.0001	<0.0001

Data are presented as mean ± standard error of the mean. The statistically significant differences between wild-type (WT) and mutant Na_v_1.2 channels were determined using one-way ANOVA, followed by Dunnett’s *post hoc* test (*P < 0.05, **P < 0.01, ***P < 0.001 and ****P < 0.0001). ND = not determined; NS = statistically not significant difference compared to WT. I_Na_ = sodium current; I_Na-P_ = persistent sodium current; *n* = number of independent experiments; t recovery = time constant of recovery from fast inactivation; V_0.5 =act_ = membrane potential for half-maximal activation; V_0.5 =inact_ = membrane potential for half-maximal inactivation.

Statistical analyses of the MD simulations were performed by first applying decorrelation analysis to extract uncorrelated data-points using time series in pymbar Python packages, then using the student’s *t*-test on the filtered data-points via the SciPy Python package.

## Results

### *SCN2A* variants and patients

We evaluated the clinical and biophysical characteristics of two *SCN2A* variants associated with early-infantile DEE (N1662D and L1657P) and performed database and literature searches identifying additional *SCN2A* variants reported as pathogenic (M1501T, M1501V, F1651C), likely pathogenic (P1658S) or pathogenic/likely pathogenic (A1659V) for biophysical studies (see the ‘Materials and methods’ section). The selection criteria were the location and mutation proximity in space relative to amino acid residue N1662 when using the published cryo-EM structure of the Na_v_1.2 channel.^16^

The N1662D variant, identified through the *SCN2A* NHS database and reported in abstract form by Takacs *et al*.,^50^ was identified by whole exome sequencing in an individual born at 34 weeks gestation. This individual had early-infantile developmental and epileptic encephalopathy, with seizure onset on Day 1 of life. Initial seizure types were focal and tonic, and EEG in the neonatal period demonstrated a burst-suppression pattern. Some initial reduction in seizure frequency was noted with phenobarbitone and phenytoin, although phenytoin was reported to increase sedation. Ultimately, seizures were resistant to all treatments tried, including sodium channel blocking (SCB) anti-seizure medication (SCB: lacosamide, oxcarbazepine, zonisamide, non-SCB: levetiracetam). She experienced frequent episodes of status epilepticus until initiation of the ketogenic diet (KD) at age 3 years of age. KD remains the most effective treatment and has significantly reduced ICU admissions. Her current epilepsy treatments are KD, lacosamide, oxcarbazepine, zonisamide and phenobarbitone. Her brain MRI at age 5 years showed severe cerebral and cerebellar atrophy, with diffusion restriction present in the bilateral symmetric medial temporal lobes, thalamus and hippocampi. Her EEG at age 6 years showed a diffusely suppressed background without interictal epileptiform activity. This child continues to experience profound developmental delays, severe hypotonia and microcephaly, is gastrostomy-fed, and has a tracheostomy.

L1657P has been identified in two patients. The first is a male, born after a normal pregnancy and well at birth. Seizures started from Day 2 of life onwards with frequent daily episodes of tonic seizures, spasms and status epilepticus, with burst-suppression pattern on the EEG (consistent with EIDEE) and normal brain MRI. Treatment with phenobarbital, levetiracetam, vigabatrin or ketogenic diet showed no effect. With phenytoin treatment, seizures and EEG improved, but daily seizures persisted. Addition of valproate, bromide or chloral hydrate had no effect on epilepsy. The child showed a severe global developmental disorder, an extreme severe form of spasticity, had no eye contact and little reaction to stimuli, and was fed with a PEG tube. The child died at age 20 months from pneumonia.

The second is a boy born at term, with onset of electroclinical and electrographic focal seizures on Day 3 of life. The epilepsy syndrome consistent with epilepsy of infancy with migrating focal seizures. On neurologic examination, he was encephalopathic and hypertonic. Seizures were resistant to phenobarbitone, levetiracetam, vigabatrin and lacosamide. An initial reduction in seizure frequency was noted with each of carbamazepine, phenytoin and clobazam, but this was not sustained. At age 4 months, seizures continue at between multiple daily and multiple hourly frequency and are often prolonged.

P1658S, identified through the ClinVar database, has been described as a likely pathogenic variant.

A1659V has been identified in a cohort of Vietnamese DEE patients and reported as pathogenic/likely pathogenic.^25^

M1501T (ClinVar) has been associated with early-infantile DEE.

M1501V identified through the SSDb database, has been reported in a male patient and described as a pathogenic *de novo* variant associated with early-infantile DEE.

F1651C has been identified as a mosaic *de novo* mutation in a patient with early-infantile DEE characterized by seizure onset at 6 weeks of age, with seizures classified as clonic and generalized tonic-clonic, showing multifocal spikes and episodes of status epilepticus. At 3 months, the patient became seizure free following phenytoin administration; however, low levels of phenytoin resulted in relapse.^24^

### The N1662D mutation disrupts fast inactivation

**Figure 1:**
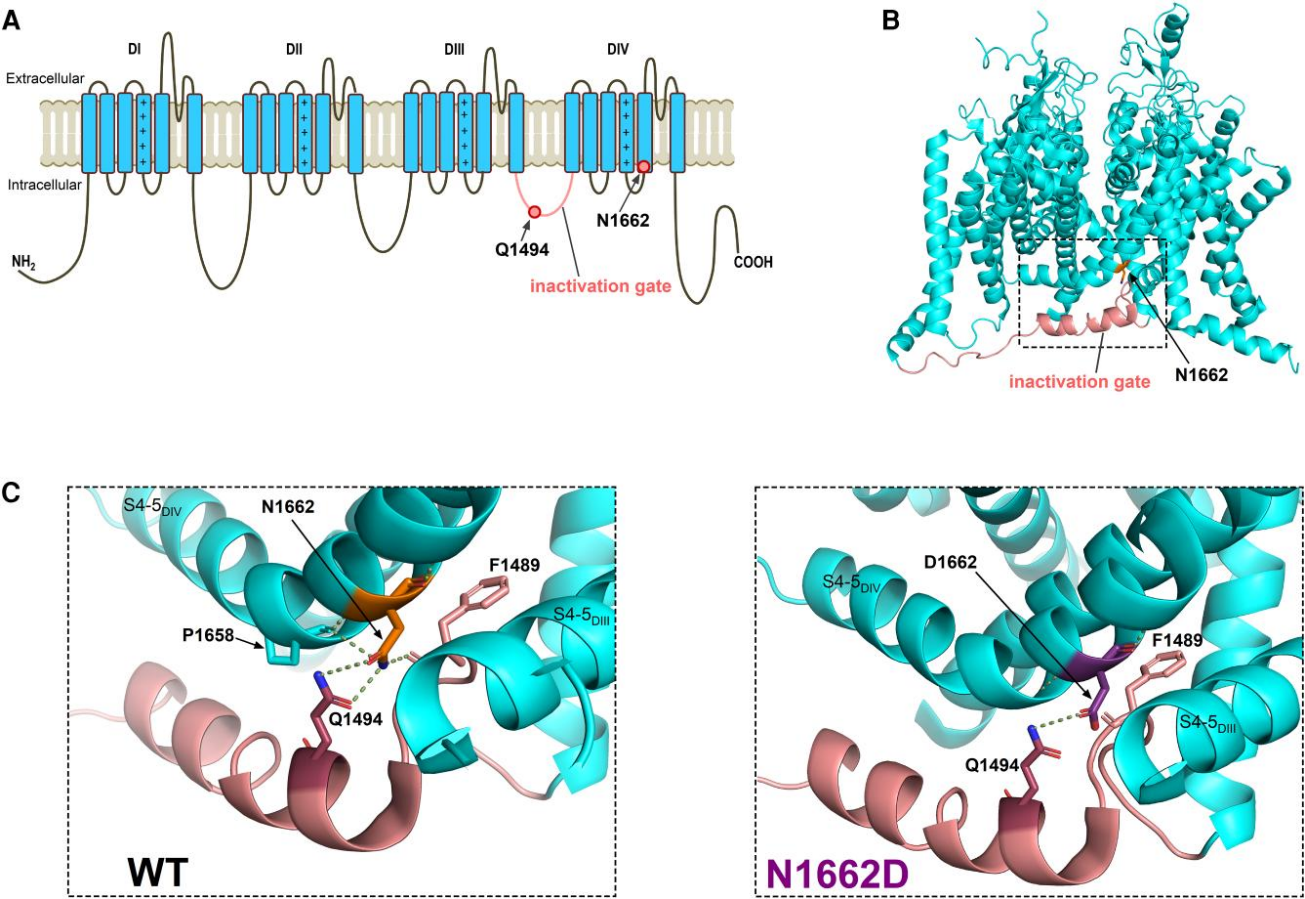
**Location of amino acid residue 1662 and impact of N1662D mutation on polar interactions.** (**A**) 2D transmembrane topology of the wild-type (WT) Na_v_1.2 channel showing domains DI-DIV and amino acid residues N1662 (cytosolic side of S5_DIV_) and Q1494 (DIII-DIV linker/inactivation gate, in pink). The positive charges in the S4 voltage-sensing segments are marked. (**B**) 3D side-view of the WT Na_v_1.2 channel (PDB accession no. 6J8E).^16^ The boxed area highlights the N1662 residue and the inactivation gate. (**C**) Hydrogen bonds (dotted lines) between N1662 and Q1494 are ambifunctional (simultaneous donor and acceptor) in the WT channel (*left*). The N1662D mutations abolishes the hydrogen donor function of residue 1662 (*right*). Note that in the WT channel, N1662D also forms polar interactions with P1658 (located in S4-5_DIV_ and F1489 (IFM motif). The N1662 to Q1494 distance measured between the electronegative nitrogen amines (in blue) and carbonyl oxygens (in red) is 2.7 Å and 3.4 Å, whereas the D1662 to Q1494 distance is 3.4 Å.

The N1662 residue is located at the cytoplasmic end of S5_DIV_, near the inactivation gate (DIII-IV linker) and the S4–5_DIII_ and S4–5_DIV_ linkers, in the proximity of the intracellular gate (Fig. 1).^16^ This suggests that the N1662D mutation may severely affect fast inactivation, in agreement with previous alanine scanning mutagenesis data.^14^ The investigation of the wild-type channel structure revealed that the N1662 residue is involved in intermolecular interactions with several adjacent residues, including Q1494 and F1489 in the D_III-IV_ linker, and P1657 in the S4-5_DIV_ linker. Remarkably, the hydrogen bonds between the N1662 and Q1494 residues are simultaneous donor and acceptor interactions that may impact the stability and orientation of the α-helices.^51^ The N1662D mutation likely disrupts hydrogen bond formation between N1662 and Q1494 (Fig. 1C).

**Figure 2:**
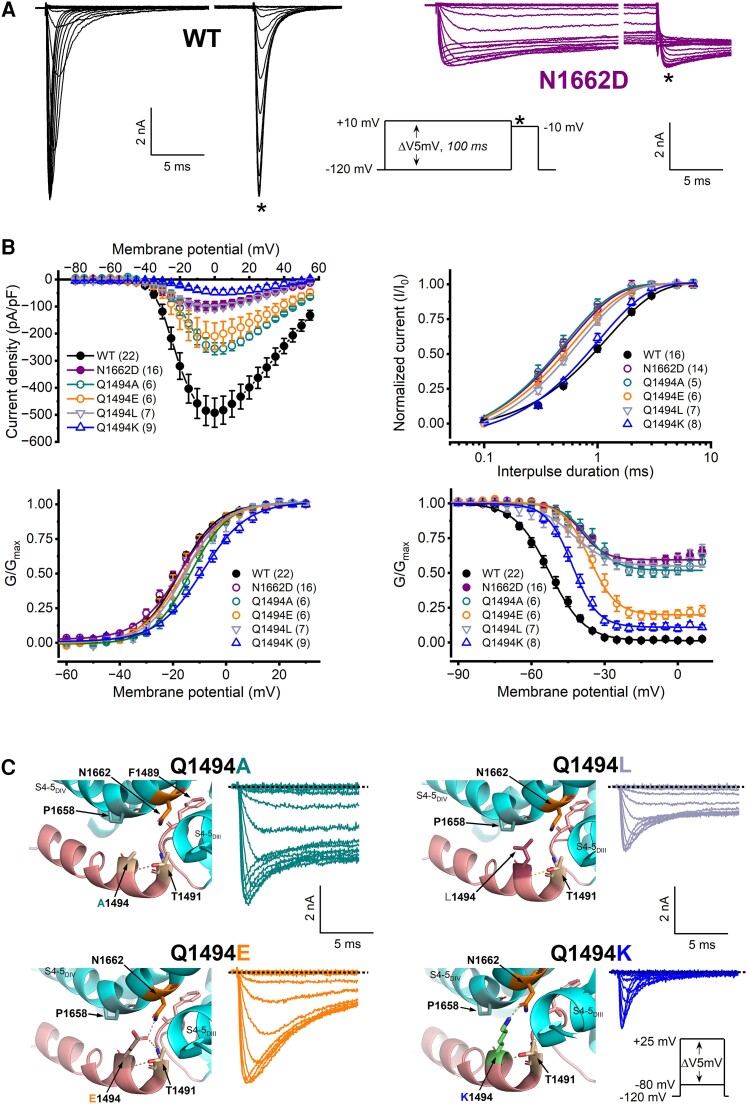
**The N1662D mutation and inactivation gate residue Q1494 mutations prevent fast inactivation.** (**A**) Representative wild-type (WT) and N1662D current traces elicited by 100-ms depolarizing (conditioning) voltage steps in 5 mV increment from a membrane potential of −120 mV, followed by a 20-ms test pulse to −10 mV to determine current availability (asterisk); *inset*: protocol. For a better resolution, an approximately 80-ms break was implemented on the *x*-axis of the WT and N1662D traces 15 ms after the onset of the inward current. (**B**) Current density-voltage relationships (*top left*). Time course of recovery from fast inactivation (*top right*). The time constants (τ-values) of recovery were obtained by non-linear fits of data to a single exponential equation (Eq. 2). Voltage dependence of activation (*bottom left*) and inactivation (*bottom right*). Activation was assessed using the inset protocol shown in **C**, whereas the protocol assessing steady-state inactivation is shown in **A**. The normalized conductance-voltage relationships are plotted as G/G_max_ values versus voltage and are referred to as activation or inactivation curves. Curves were obtained by non-linear least-squares fits of data to Boltzmann equations (Eq. 1, ‘Materials and methods’ section). In all panels, the number of experiments, *n*, is shown in parentheses. The parameters of the fits and the statistical evaluation are included in Table 1. (**C**) Mutations of the inactivation gate residue Q1494 prevent or slow fast inactivation. *Top*: Enlarged views of the S5_DIV_ and DIII-DIV linker (inactivation gate) residues, showing altered polar interactions between N1662 and A1494, E1494, L1494 or K1494 relative to Q1494 (WT; see Fig. 1). *Bottom*: Representative WT and mutant I_Na_ traces elicited from a holding potential of −120 mV by 40-ms depolarizing voltage steps in 5 mV increment (*inset*: voltage protocol). Note that only the first 15 ms of the current traces are shown. The biophysical properties of Q1494A, Q1494E, Q1494L and Q1494 channels relative to WT are shown in **B** and Table 1.

To determine the impact of the N1662D mutation on the biophysical properties of the channel, we transiently expressed the wild-type or the N1662D Na_v_1.2 channel variants in CHO cells and recorded whole-cell sodium currents (I_Na_) using the voltage clamp technique. Relative to wild-type, the N1662D mutation caused a 5-fold reduction of the I_Na_ density and almost completely prevented inactivation, resulting in a relatively small decline of the inward peak I_Na_ amplitude at the end of 100-ms depolarizations (Fig. 2A and B and Table 1). Relative to wild-type, the voltage dependence of N1662D activation was unchanged; however, the voltage dependence of the inactivated N1662D current fraction exhibited a 12-mV shift towards depolarized potentials (Fig. 2B and Table 1). Such a positive shift of the inactivation curve may predispose to enhanced neuronal excitability.^27^ On the other hand, the lack of sodium channel inactivation could lead to a sustained depolarization of the membrane potential and prevent action potential firing in neurons.^52,53^ Recovery from inactivation was faster for the N1662D variant relative to wild-type (Fig. 2B and Table 1). This may reflect a reduced affinity of the inactivation gate receptor for the IFM motif due to a non-absorbing inactivation state from which recovery is faster.^54^

### Inter-helical hydrogen bonds between N1662 and Q1494 are essential for fast inactivation

We hypothesized that the hydrogen bonds between the N1662 and Q1494 residues are critical determinants of the fast inactivation gating mechanism. To this end, we mutated the Q1494 residue to amino acids with neutral, acidic or basic side chains and examined the mutant channels biophysically. We predicted that the substitution of the neutral, polar Q1494 residue with a neutral, non-polar A or L residue would destabilize or abolish the fast inactivated state, consistent with the absence of hydrogen bonds between N1662 and A1494 or L1494. However, the substitution of Q1494 to acidic E or basic K residue should still permit the formation of a single hydrogen bond between N1662 and E1494 or K1494 and enable the development of fast inactivation to some extent.

The Q1494A and Q1494L mutations substantially prevented fast inactivation during depolarizing test pulses and resulted in biophysical properties very similar to those of the N1662D variant. The Q1494E mutant exhibited slowly decaying currents, whereas the Q1494K retained significant inactivation (Fig. 2B and C). Relative to wild-type, all four engineered mutations resulted in decreased current densities, faster recoveries of the inactivated fraction and large depolarizing shifts of the inactivation curve (Fig. 2B and C and Table 1), but had limited effects on activation, resulting in small shifts of the activation curve (Fig. 2B and C and Table 1). These results demonstrate that hydrogen bond interactions between the N1662 and Q1494 residues represent critical molecular prerequisites for the development of fast inactivation in Na_v_1.2 channels.

### Molecular dynamics simulations reveal a reduced stability of the inactivated state in mutant channels

To confirm the structural basis underlying the disrupted fast inactivation in N1662D channels, we conducted MD simulations on the inactivated Na_v_1.2 structure. In over five replicates of 1 µs simulations, we assessed recovery from the fast-inactivated state by observing the relative stability of the DIII-IV linker and the binding of the IFM motif to its pocket.

**Figure 3:**
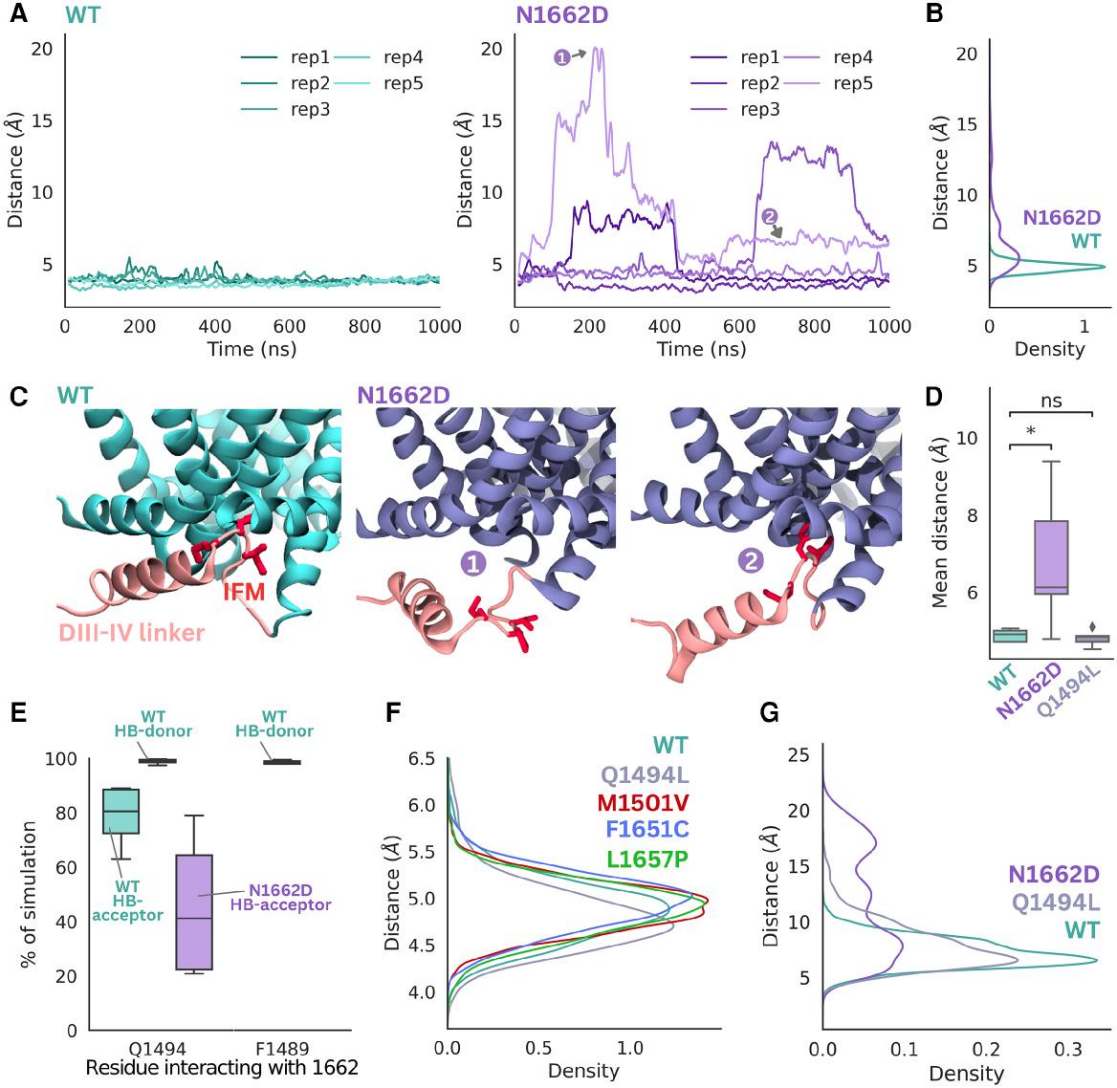
**Stability of IFM-motif binding affected by different mutations compared to wild-type shown in molecular dynamics simulations**. (**A**) Distance between the IFM motif and its binding pocket (defined as the center of mass of F1489 and the center of mass of residues 1659, 1663 and 1769) determined across the time course of each molecular dynamics (MD) trajectory for wild-type (WT; cyan) and N1662D (purple). Each of the five replicates plotted as a separate line. (**B**) Density plots showing differences in the distributions of distance between IFM motif and its binding pocket for WT and N1662D. (**C**) Representative snapshots taken from the trajectories showing the stably bound IFM motif in WT and the dissociation of the IFM motif and DIII-IV helix in N1662D at different time points (marked as 1 and 2). (**D**) The distance between the IFM motif and its receptor were averaged for each of the five replicates for WT, N1662D and Q1494L and shown as a box plot. This distance significantly increased in the N1662D (*P* = 0.044) and was unchanged in Q1494L (*P* = 0.57). (**E**) Percentage of simulation where hydrogen donor and acceptor interactions were detected between residue 1662 and surrounding residues in WT (cyan) compared to N1662D (purple). (**F**) Distributions of distance between IFM motif and its binding pocket for other variants, Q1494L (grey), M1501V (red), F1651C (blue) and L1657P (green), were very similar to WT, as seen in unbiased MD simulations. (**G**) REST2 simulations were able to capture dissociation of the IFM in both N1662D and Q1494L.

The N1662D mutant showed pronounced differences in simulations compared to wild-type, displaying spontaneous dissociations of the DIII-IV linker within 1 µs in three of five replicates, whereas the IFM motif of the wild-type channel remained bound across all replicates (Figs 3A–C and Supplementary Videos 1 and 2). Distances between the centre of mass of IFM motif residues and the centre of mass of its binding site residues were significantly different between wild-type and the N1662D mutant (Fig. 3D), suggesting that the mutation perturbs binding of the IFM motif. In wild-type, the ambifunctional hydrogen bonding configuration between Q1494 and N1662 was maintained throughout simulations (Fig. 3E), contributing to stabilization of the bound DIII-IV linker in the inactivated state. Hydrogen bond donor interactions between N1662 and Q1494 were present in almost 100% of wild-type simulations. Notably, N1662 forms hydrogen bond donor interactions with the F1489 backbone (IFM motif residue), which likely further increases the binding stability of the DIII-IV linker. The mutation to D1662 results in loss of hydrogen bond donor interactions, due to lack of the nitrogen atom, and contributes to instability in binding of the linker (Fig. 3E).

To investigate the contributions of Q1494 to the stabilization of IFM motif binding, we simulated the Q1494L mutant. Surprisingly, unlike N1662D, Q1494L was very similar to wild-type, with the IFM motif remaining stably bound across replicates (Fig. 3D and F). Although the Q1494L mutation also disrupts the 1662–1494 hydrogen bonding network, the N1662 can still form a hydrogen bond with F1489, similar to wild-type (Fig. 3E), thus reducing the propensity for IFM motif dissociation.

Given that the Q1494L mutation likely has a more subtle influence on the stability of the IFM motif than N1662D that was challenging to capture within the time scale of our 1 µs unbiased MD simulations, we used an enhanced sampling method (REST2) to better examine the stability of the IFM motif in the two mutants. For N1662D, the distribution of the distance between the IFM motif and its receptor shows two peaks greater than 10 Å, indicating a greatly reduced binding affinity compared to wild-type (Fig. 3G). The Q1494L variant shows a more subtle effect where the distances are slightly greater than the wild-type, suggesting a decreased binding stability of the IFM (Fig. 3G). Overall, this highlights how different mutations in the DIII-IV linker can have varying effects on IFM binding and, therefore, inactivation, with N1662D having a stronger effect than Q1494L.

Apart from unbinding of the IFM motif, dissociation of other regions of the DIII-IV linker was also captured in the N1662D equilibrium simulation. Notably, the helical portion of the DIII-IV linker is observed to fluctuate in its orientation and move away from the pore gate (Fig. 3C, right two panels). Thus, we hypothesized that residues in the DIII-IV linker, upstream of the IFM motif, are also responsible for binding to the S4-S5DIV linker and are also important for the inactivation mechanism. Overall, MD simulations demonstrate that interactions between N1662 and Q1494 play an important role in the spatial organization of the DIII-IV linker and binding of the IFM motif to its receptor site.

### Membrane voltage responses depend on the N1662D I_Na_ amplitude in axon initial segment hybrid neurons

**Figure 4:**
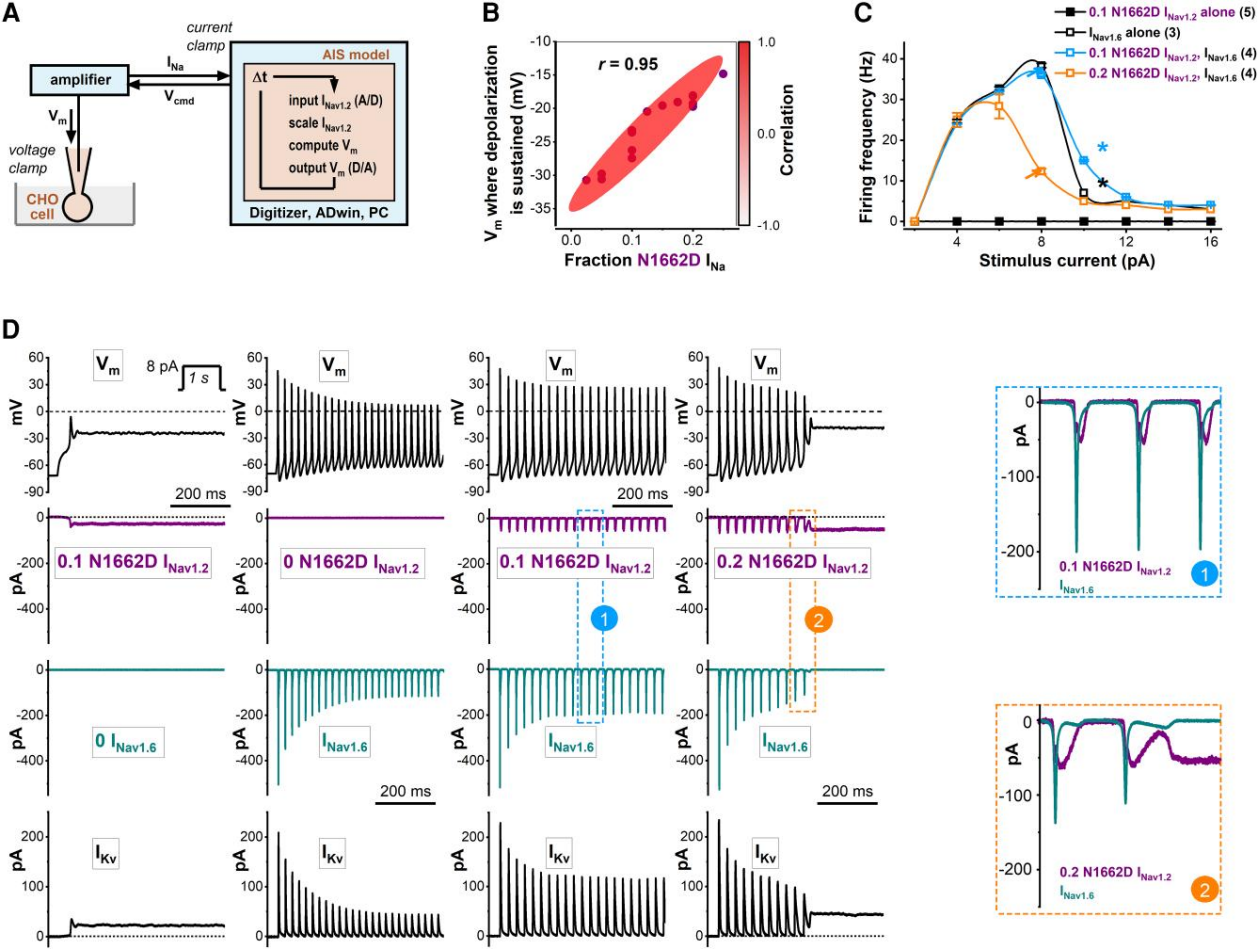
**Excitability of hybrid neurons incorporating wild-type or N1662D Na_v_1.2 channels in dynamic action potential clamp (DAPC) experiments**. (**A**) Schematic representation of the DAPC configuration, consisting of a mammalian (Chinese Hamster Ovary, CHO) cell transiently expressing wild-type (WT) or N1662D Na_v_1.2 channels and an axon initial segment (AIS) model. The membrane potential (V_m_) is calculated in real-time (using a time step, Δt, of 140 kH to update the input and output values) and applied as a voltage clamp command to the CHO cell membrane to elicit WT or N1662D Na_v_1.2 current (I_Na_). This ‘external’ I_Na_ is scaled and implemented in the model neuron. Unless mentioned otherwise, the *in silico* Na_v_1.6 conductance is set to zero. (**B**) Relationships between the magnitude of the implemented N1662D (I_Na_ fraction) and the mean V_m_ where sustained depolarization develops. The shaded red area represents a strong positive correlation (*r* = 0.95); the number of individual experiments, *n* = 14. (**C**) Input-output relationships in the hybrid model neuron incorporating external N1662D I_Na_ alone (I_Nav1.2_, filled black squares), *in silico* WT Na_v_1.6 current alone (I_Nav1.6_, open black squares), I_Nav1.6_ and 0.1 external N1662D I_Nav1.2_ (open blue squares), or I_Nav1.6_ and 0.2 external N1662D I_Nav1.2_ (open orange squares). Relative to hybrid neurons incorporating 0.2 N1662D I_Nav1.2_ and I_Nav1.6_, neurons with an increased N1662D I_Nav1.2_ fraction show higher firing activity (orange and blue arrows, respectively). Note that firing activity can be higher in hybrid cells implementing 0.1 N1662D I_Nav1.2_ and I_Nav1.6_ relative to I_Nav1.6_ alone (blue and black stars, respectively). (**D**) Relationships between the V_m_ and selected membrane current components of the hybrid model neuron, showing representative V_m_ changes (traces at *top*) and associated scaled external input N1662D I_Nav1.2_, *in silico* I_Nav1.6_ (downward deflections) and *in silico* voltage-gated potassium currents (I_Kv_, upward deflections). From *left*: N1662D I_Nav1.2_ alone (purple traces) elicits sustained depolarization. Note the associated sustained non-inactivating I_Nav1.2_ and the depressed I_Kv_ (black traces). *In silico* I_Nav1.6_ alone (dark cyan traces) is associated with sustained firing. In the presence of 0.1 or 0.2 I_Nav1.2_, *in silico* I_Nav1.6_ differentially enhances or reduces firing. Dashed lines indicate the 0-mV membrane potential (V_m_) level; dotted lines indicate the zero-pA current level; *inset* step current stimulus; the first 500 ms of the 1 s traces of V_m_ and current traces are shown. See relationships between the inward currents associated with action potential firing from the boxed areas marked with ‘1’ and ‘2’.

**Figure 5:**
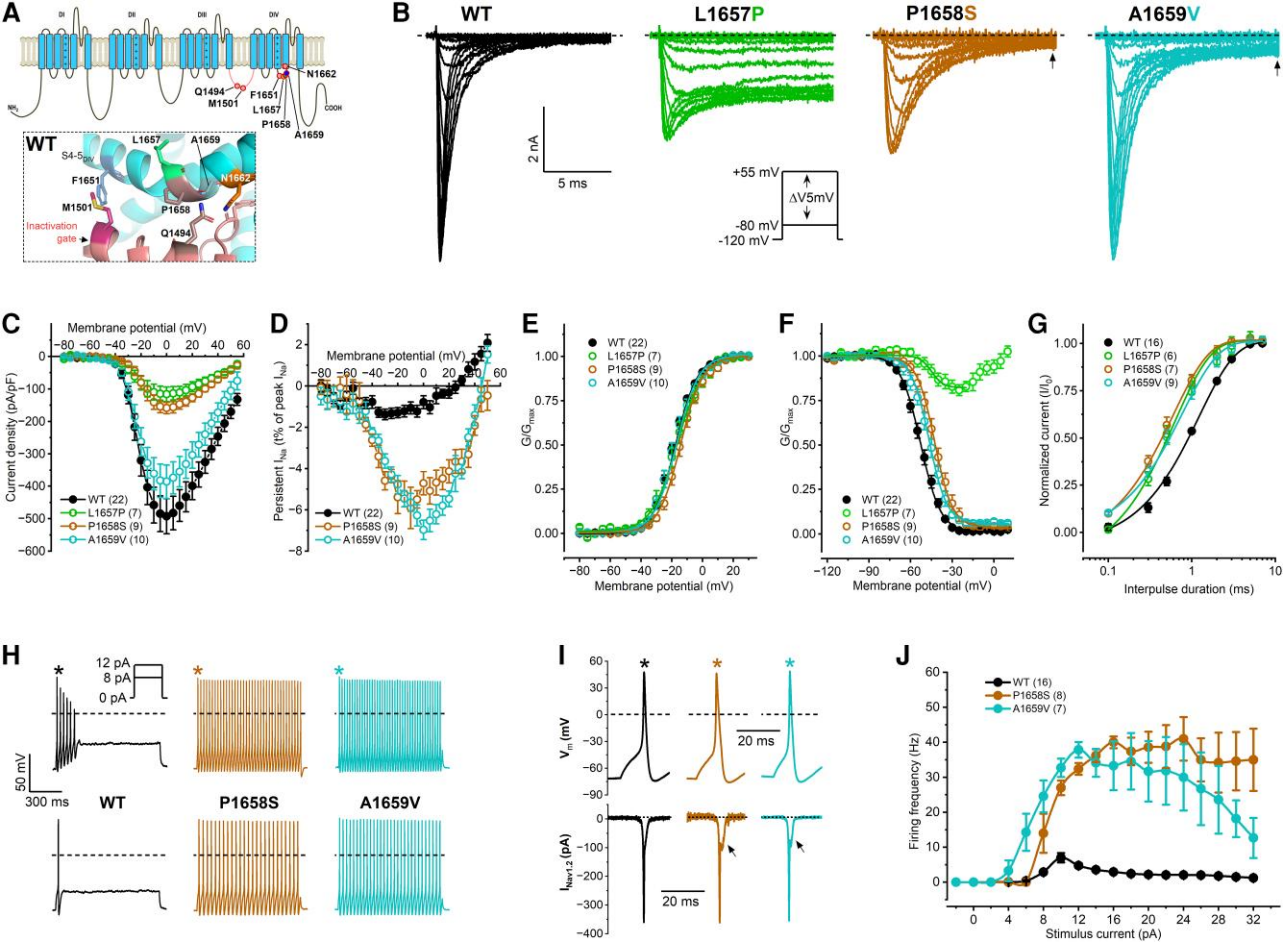
**Biophysical properties and functional impact of the wild-type and pathogenic L1657P, P1658S and A1659V variants.** (**A**) Schematic representation of the assessed mutations relative to the N1662 residue. Zoomed-in cartoon representation of the S4-5D_IV_ linker structure in the wild-type (WT). For additional details, see Fig. 1A. (**B**) Representative WT and mutant I_Na_ traces elicited from a holding potential of −120 mV by 40-ms depolarizing (conditioning) voltage steps in 5 mV increment (*inset*: voltage protocol). The L1657P mutation produces non-inactivating current, whereas the P1658S and A1659V mutations result in a large persistent current relative to WT (arrows). Dashed lines indicate zero current level; only the first 14 ms of the current traces elicited in the voltage range between −80 and +10 mV are shown. (**C**) Current density-voltage relationships. (**D**) Persistent inward I_Na_-voltage relationships. (**E**) Voltage dependence of activation (**F**) Voltage dependence of steady-state inactivation. (**G**) Recovery from fast inactivation. Data in **E–G** were obtained and fitted as described in Fig. 1. See parameters of the fits and statistical evaluation in Table 1. (**H**) Representative action potential traces in hybrid neurons incorporating transiently expressed WT, P1658S, or A1659V Na_v_1.2 currents in dynamic action potential clamp (DAPC) experiments. Traces elicited using 8 or 12 pA step stimuli are shown; dashed lines indicate zero V_m_ level. (**I**) Action potentials (corresponding to the action potentials marked by asterisks in **H**) and associated WT (black), P1658S (brown) or A1659V (turquoise) I_Nav1.2_ traces on expanded time scale; arrows indicate the increased persistent current component relative to WT; dotted lines indicate zero current level. (**J**) Input-output relationships. Firing frequencies of mutants relative to WT were assessed using two-way ANOVA followed by Dunnett’s *post hoc* test (*P < 0.05). Data are mean ± standard error of the mean; *n*, number of individual experiments shown in parentheses.

It is plausible that in the axon initial segments of patients carrying non-inactivating heterozygous Na_v_1.2 variants such as N1662D, firing activity is modulated by the expression level of the mutant Na_v_1.2 channel protein. To evaluate the impact of the N1662D channels on neuronal excitability, we characterized the voltage responses of the AIS hybrid cell model incorporating wild-type or N1662D I_Na_ in DAPC experiments (Fig. 4A). Hybrid neurons incorporating wild-type I_Na_ showed firing activity with a characteristic bell-shaped input-output relationship (Fig. 5J). To mimic the reduced expression level of N1662D channel relative to wild-type (Fig. 2A), the inward peak amplitude of the implemented N1662D I_Na_ was systematically reduced. For example, a 5-fold lower N1662D expression relative to wild-type corresponded to a 0.2 external I_Na_ fraction (∼80 pA N1662D I_Na_). Hybrid neurons incorporating N1662D I_Na_ alone did not fire action potentials during the step stimuli but showed sustained depolarization of the membrane potential due to the non-inactivating N1662D I_Na_ (Fig. 4C and D and Supplementary Fig. 1). Sustained V_m_ depolarization could be elicited with a range of N1662D I_Na_ amplitudes and its magnitude was positively correlated with the implemented N1662D I_Na_ fraction (Fig. 4B).

Hybrid neurons incorporating *in silico* I_Nav1.6_ alone elicited repetitive firing showing accommodation (Figs 4C and D). We varied the amplitude of the external N1662D Na_v_1.2 current (I_Nav1.2_) in a hybrid neuron in the absence or presence of ‘co-expressed’ *in silico* Na_v_1.6 current (I_Nav1.6_). Surprisingly, implementing 0.1 N1662D I_Nav1.2_ and *in silico* I_Nav1.6_ caused increased excitability, less accommodation, action potentials with increased amplitude, and more efficient action potential repolarization relative to I_Nav1.6_ alone (Fig. 4C and D). However, a further increase of the non-inactivating I_Nav1.2_ fraction to 0.2 in the presence of I_Nav1.6_ led to a decrease in firing and transitioning of the membrane potential to a sustained depolarized value (Fig. 4C and D). These experiments also demonstrate that membrane potential changes are associated with dynamic and complex changes of the current components in the hybrid neuron, contributing to the shifts in balance between inward (I_Na_) and outward potassium (I_Kv_) currents (Fig. 4D). We investigated the contribution of external wild-type I_Na1.2_ and 0.1 N1662D I_Nav1.2_ to total sodium entry with or without I_Nav1.6_. The results indicate that in the presence of *in silico* I_Nav1.6_, N1662D I_Nav1.2_ increases sodium influx during single action potentials compared to wild-type I_Nav1.2_, which correlates with increased I_Kv_. Notably, I_Kv_ in hybrid neurons with 0.1 N1662D I_Nav1.2_ alone fails to initiate repolarization, resulting in sustained depolarization in DAPC experiments (Supplementary Fig. 4).

### Na_v_1.2 missense mutations affecting fast inactivation gating machinery result in hyperexcitability

The S4-5_DIV_ and DIII-IV linkers may undergo relatively large movements during fast inactivation gating, thus missense mutations affecting residues located in these regions are expected to impact channel function severely. We evaluated the biophysical characteristics and the impact on neuronal excitability of several DEE-associated missense mutations located in the inactivation gate (M1501T, M1501V) or S4-5DIV linker (F1651C, L1657P, P1658S, A1659V). The clinical features and treatment responses of individual patients are summarized in the Supplementary material.

The L1657P, P1658S and A1659V mutations are localized near the N1662 residue (Fig. 5A). Voltage clamp experiments revealed reduced current density and a depolarizing shift of the activation curve for P1658S, whereas the current densities and the activation curve for A1659V remained unchanged relative to wild-type (Fig. 5C and E and Table 1). For both P1658S and A1659V, the inactivation curves shifted towards depolarized voltages, and the recoveries from fast inactivation were faster (Fig. 5F and G and Table 1). Although P1658S and A1659V retained fast inactivation, these variants exhibited a very large inward persistent current (I_Na-P_) relative to wild-type, corresponding to ∼6% of the transient peak I_Na_ (Fig. 5D and Table 1). I_Na-P_ can have a considerable impact on excitatory neuronal function, influencing repetitive firing and facilitating hyperexcitability.^55^ Relative to wild-type, the P1658S or A1659V I_Na_ resulted in a significantly higher firing activity in DAPC experiments, associated with an increased I_Na-P_ during the repolarization phase (Fig. 5H–J).

The L1657P variant resulted in a large, non-inactivating I_Na_, (Fig. 5B). The biophysical properties of the L1657P variant were similar to those of N1662D (Figs 5C–G and Table 1). It is likely that the L to P mutation breaks or kinks the S4-5_DIV_ α-helix and disrupts the formation of the hydrogen bonds needed to maintain the stability of the protein structure. In DAPC experiments, hybrid neurons incorporating L16757P I_Na_ alone showed sustained depolarization. In the presence of *in silico* I_Nav1.6_, a relatively small-amplitude external L16757P I_Na_ led to a transient increase of firing activity followed by sustained membrane depolarization, similar to N1662D I_Na_ (Supplementary Fig. 2).

The 3D structure of the Na_v_1.2 channel suggests that in addition to hydrogen bond interactions shown in Figs 1 and 2, additional interactions may also contribute to the stability of the surface exposed, highly mobile inactivation gate. The residues M1501 and F1651, located on the DIII-IV linker and the S4-S5_DIV_ linker, respectively (Fig. 5A and Supplementary Fig. 3), are thought to form a critical aromatic-sulfuric interaction, given that the F1651A mutation is known to result in a slow and incomplete fast inactivation.^14^ Mutations of the M1501 or F1651 residue may affect fast inactivation by destabilizing the binding of the IFM motif; however, the effect may be less pronounced relative to those caused by N1662D. In MD simulations of the M1501V and F1651C mutations, we observed some instability between the DIII-IV linker and the S4-S5_DIV_ linker; however, dissociation of the IFM motif could not be revealed with the IFM motif consistently bound throughout 1 µs simulations (Fig. 3F). Supplementary Fig. 3 shows the biophysical consequences of the pathogenic F1651C, M1501V and M1501T mutations and their impact on neuronal firing. As expected, these mutations also severely affected the voltage dependence and the recovery from fast inactivation and increased the inward I_Na-P_ component relative to wild-type. DAPC experiments provided a rapid prediction of the neuron-scale phenotypic consequences of these three variants, resulting in increased action potential firing of the hybrid neuron relative to wild-type (Supplementary Fig. 3). The gain-of-function effects are consistent with the reported severe clinical phenotypes of these variants.

## Discussion

In sodium channels, the binding of the hydrophobic IFM motif to the inactivation receptor site represents an essential conformation change leading to fast inactivation.^4-6,56^ Inactivation can be prevented by destroying the inactivation gate using internally perfused pronase,^57^ preventing the outward movement of the S4_DIV_ using extracellular site 3 toxins,^58^ or mutating residues of the IFM motif or the inactivation receptor.^13,14^ In this study, we demonstrate that fast inactivation of the Na_v_1.2 channel can also be prevented by missense mutations associated with early-infantile DEE.

Recent high-resolution structure data demonstrate that the IFM motif binds to a receptor site located on pore regions located outside of the pore-lining helices.^18,20^ The rate of inactivation is largely aligned with the relatively slow movement of S4_DIV_, after which the IFM motif may bind through a weakly voltage-dependent step to its receptor.^7^ Despite the proposed molecular models of fast inactivation gating,^15,18,19^ the details of the interactions between the residues involved are not fully understood.

### The influence of disrupted inactivation on channel function and neuronal excitability

Genetic diagnoses and functional-clinical evaluation of rare missense Na_v_1.2 variants revealed that pathogenic mutations can occur at various channel regions and can lead to gain-of-function or loss-of-function.^24^ Electrophysiological evaluation of Na_v_1.2 variants confirmed gain-of-function produces early-infantile DEE.^27,59^ Here, we performed voltage clamp and dynamic action potential clamp analyses to evaluate the biophysical and neurophysiological consequences of missense Na_v_1.2 variants associated with early-infantile DEE. Seven mutations in channel sequences significantly involved in fast inactivation gating were studied.

Five variants demonstrated the expected gain-of-function. The F1651C, P1658S, A1659V, M1501V and M1501T variants shifted the voltage dependence of steady-state inactivation to more positive membrane potentials and resulted in fast recovery from inactivation. The mutant currents displayed enhanced inward I_Na-P_ with an amplitude between 3% and 6% of the transient inward peak I_Na_ because of incomplete or delayed fast inactivation. In DAPC experiments, each variant caused hyperexcitability when implemented in an excitatory hybrid neuron model.

Remarkably, the N1662D and L1657P variants did not result in gain-of-function, but instead resulted in non-inactivating currents, highlighting a novel mechanism for *SCN2A*-related early-infantile DEE. Given that sodium current inactivation represents a crucial aspect of neuronal excitability regulation, the lack of inactivation can lead to a prolonged depolarized state and affect the ability of neurons to fire action potentials effectively. DAPC experiments demonstrate that in the absence of other types of sodium conductances, N1662D or L1657P I_Na_ invariably results in the sustained depolarization of the hybrid neuron model.

However, it is important to note that both the N1662D and the L1657P variant showed reduced current densities relative to wild-type in transfected mammalian cells. This suggests that the impact on neuronal excitability may vary depending on several factors, including the variability in *SCN2A* allelic expression, the contribution of other neuronal sodium channels, and/or other cellular mechanisms. When implementing N1662D or L1657P I_Na_ with ∼10-fold reduced density relative to the enabled *in silico* Na_v_1.6 conductance, the mutant current triggers increased excitability in DAPC experiments. It is likely that this effect is due to the mutant current causing a relatively small depolarization of the membrane and/or contributing inward current during the activation of other types of neuronal sodium channels. In the context of SCN2A variants, overall gain-of-function can occur despite decreased current density if the mutation alters channel kinetics or other functions, leading to neuronal hyperexcitability or abnormal firing patterns.

### Structural determinants of fast inactivation in Na_v_1.2 variants

MD simulation studies were performed using a Na_v_1.2 structure with an activated S4_DIV_ and IFM motif bound to its pore-module receptor^16^ from which the IFM motif spontaneously dissociates in the N1662D mutant, reflecting a mutation-disrupted inactivation state. In agreement with the functional data, MD simulations revealed an increased distance between D1662 and Q1694 relative to wild type channel, indicating that the N1662D mutation reduces the affinity of the IFM motif to the DIVS4-S5 linker. The data also suggest that multiple hydrogen bond formation between the N1662 and Q1494 side chains is essential for the stability of the local DIII-IV linker region in the inactivated state. We hypothesize that instability of the linker is responsible for the accelerated rates of recovery from fast inactivation in the N1662D variant (Fig. 2B) and contributes to overall disruption of the inactivation mechanism. This outcome substantiated mutagenesis experiments demonstrating that fast inactivation is also prevented in the Q1494A or Q1494L Na_v_1.2 channel variants, whereas the Q1494E or Q1494K variants enable an incomplete inactivation characterized by an enhanced I_Na-P_ relative to wild-type.

Although MD simulations were able to reveal significant differences in the distance between the IFM and its receptor pocket in the N1662D variant relative to wild-type, the differences for Q1494L were more subtle, only clearly showing in enhanced sampling MD simulations. Similarly, the respective distance for the L1657P variant was unchanged relative to wild-type (Fig. 3F). It is likely that both Q1494L and L1657P allow some hydrogen bonds to remain and thus have a weaker influence on the affinity of the IFM motif to its receptor. The mutational effects of M1501V/T and F1651C are less clear. We do not see strong deficiency of fast inactivation in electrophysiology experiments, which is recapitulated in MD simulations where the IFM-motif does not dissociate. The M1501-F1651 interactions may have more complex structural implications that control the voltage dependence of inactivation.

### *SCN2A* variant interpretation and challenges for treatment

Given the size of the *SCN2A* gene, it holds immense potential for genetic variations associated with significant phenotypic heterogeneity. The actual number of distinct *SCN2A* mutations observed in human populations continues to grow as more individuals are sequenced and studied. The extensive research on genetic epilepsies, including *SCN2A*, enhances our understanding of *SCN2A* disorders, enables the identification of novel mechanisms and therapeutic targets, and promises better personalized treatment strategies for affected individuals.

Currently, single-cell voltage clamp techniques are commonly used to characterize the functional properties of pathogenic Na_v_1.2 variants. However, predicting the overall impact of Na_v_1.2 channel mutations on neuronal excitability is challenging due to the complex interplay of gain- or loss-of-function characteristics in individual biophysical traits. Our research demonstrates that the interpretation of voltage clamp data can be significantly strengthened by DAPC analysis.^27^ Future research should evaluate the effects of specific ion channel mutations across neuron models of varying complexity. This approach would not only advance DAPC methodology but also offer more refined predictions of functional outcomes compared to the relatively simplistic AIS compartment model. While the advantages of DAPC are evident, conventional voltage clamp assays failed to identify the underlying dysfunction mechanisms in N1662D Na_v_1.2 and L1657P Na_v_1.2 due to their reduced expression levels in the heterologous expression system. Consequently, these variants would have been erroneously classified as loss-of-function.

Furthermore, our study demonstrates that the availability of high-resolution sodium channel structures significantly enhances the understanding of the molecular basis of ion channel dysfunction. We identified key molecular interactions that are critical for the fast inactivation process in Na_v_1.2 channels. While this is important from a fundamental science perspective, the findings have therapeutic implications. Traditional sodium channel blockers, such as carbamazepine or phenytoin, may be less effective for targeting non-inactivating channels. Gain-of-function mutations are a particularly attractive target for antisense oligonucleotide (ASO) therapy, as reducing the RNA transcript and subsequent functional protein to restore physiological function can correct gene-driven pathologies.^60^ Although ASO gene therapy offers hope for a revolutionary cure for infantile *SCN2A* DEE, it would not address those patients with non-inactivating Na_v_1.2 variants. However, a combined ASO strategy involving mutant allele-specific downregulation alongside upregulation of the wild-type allele holds potential for restoring a balanced expression of Na_v_1.2 channels, thereby alleviating the effects of *SCN2A* haploinsufficiency. Additionally, exploring novel or existing drugs capable of modifying Na_v_1.2 channel inactivation offers an alternative approach for targeting non-inactivating Na_v_1.2 channels.

The anti-seizure sodium channel blockers phenytoin, carbamazepine and lamotrigine bind to receptor sites located inside the central cavity of the pore^61^ and have a higher affinity to the fast inactivated state than to the resting state.^62^ In addition to these actions, the slow inactivation of the channel may be affected by phenytoin^63^ and the voltage sensors may also be implicated in phenytoin binding.^64^ The lack of fast inactivation in N1662D and L1657P channels suggests that the affinity to common anti-seizure sodium channel blockers may be reduced. Indeed, the individual with the N1662D variant had little benefit from most sodium channel blockers used. However, given that neither the N1662D nor the L1657P mutation alters the voltage dependence of activation, it is also plausible that the sodium channel blocking effect of phenytoin is unaffected or relatively less affected. Supporting this latter hypothesis, in the patients with the L1657P variant (and to a lesser extent the individual with the N1662D variant), phenytoin treatment reduced seizures, although frequent seizures persisted. Future experiments are needed to clarify the effect of the commonly used anticonvulsants on non-inactivating sodium channel variants. The selective late (persistent) sodium current inhibitor GS-967^65^ efficiently suppressed the non-inactivating I_Na_ component in CHO cells transiently expressing L1657P Na_v_1.2 channels (Supplementary Fig. 5). Notably, GS-967 concentrations resulting in half-maximal inhibition of the non-inactivating component did not inhibit the transient I_Na_ component, suggesting that agents exhibiting similar properties could represent promising candidates for future development. Targeting the non-inactivating current with ranolazine^66^ or using open channel blockers such as propafenone^19^ may also be efficient.

Identifying novel mechanisms of ion channel dysfunction underscores the ongoing need to explore innovative therapies and compounds capable of precisely targeting these mechanisms. The neurophysiological consequences of non-inactivating Na_v_1.2 variants need to be investigated in various *in vitro* and *in vivo* models. Establishing and assessing induced pluripotent stem cell (iPSC)-derived neuronal models for the N1662D and L1657P variants could further clarify their specific functional and transcriptomic *in vitro* phenotypes. Additionally, creating mouse models carrying the corresponding non-inactivating Na_v_1.2 variants may replicate the severe DEE symptoms observed in patients and enable effective testing of therapeutic strategies, with implications for translation into clinical practice.

In conclusion, we demonstrate that the N1662-Q1494 interaction is essential for maintaining the binding affinity of the IFM motif to its receptor in fast inactivated Na_v_1.2 channels. Our data have implications for elucidating fast inactivation of Na_v_1.2 channels and interpreting the impact of Na_v_1.2 variants on neuronal excitability. By working at multiple scales, we can connect the clinical symptoms experienced by DEE patients to their cellular and molecular basis, while elucidating fundamental aspects of sodium channel function. Non-inactivating Na_v_1.2 channel variants can lead to sustained depolarization of pyramidal neurons; however, the overall effect on excitability may depend on the expression level of the channel protein.

## Supplementary Material

awae213_Supplementary_Data

## Data Availability

All unique constructs and datasets generated during and/or analysed during the current study are available from the Lead Contact on reasonable request from any qualified investigator for purposes of replicating procedures and results.

## References

[1] Scheffer IE , Berkovic S, Capovilla G, et al ILAE classification of the epilepsies: Position paper of the ILAE commission for classification and terminology. Epilepsia. 2017;58:512–521. 28276062 10.1111/epi.13709 PMC5386840

[2] Epi KC , Phenome/Genome E, Allen P, et al De novo mutations in epileptic encephalopathies. Nature. 2013;501:217–221. 23934111 10.1038/nature12439 PMC3773011

[3] Sanders SJ , Campbell AJ, Cottrell JR, et al Progress in understanding and treating *SCN2A*-mediated disorders. Trends Neurosci. 2018;41:442–456. 29691040 10.1016/j.tins.2018.03.011 PMC6015533

[4] Ulbricht W . Sodium channel inactivation: Molecular determinants and modulation. Physiol Rev. 2005;85:1271–1301. 16183913 10.1152/physrev.00024.2004

[5] Catterall WA . From ionic currents to molecular mechanisms: The structure and function of voltage-gated sodium channels. Neuron. 2000;26:13–25. 10798388 10.1016/s0896-6273(00)81133-2

[6] Goldin AL . Mechanisms of sodium channel inactivation. Curr Opin Neurobiol. 2003;13:284–290. 12850212 10.1016/s0959-4388(03)00065-5

[7] Ahern CA . What activates inactivation? J Gen Physiol. 2013;142:97–100. 23858004 10.1085/jgp.201311046 PMC3727303

[8] Aldrich RW , Corey DP, Stevens CF. A reinterpretation of mammalian sodium channel gating based on single channel recording. Nature. 1983;306:436–441. 6316158 10.1038/306436a0

[9] Hodgkin AL , Huxley AF. A quantitative description of membrane current and its application to conduction and excitation in nerve. J Physiol. 1952;117:500–544. 12991237 10.1113/jphysiol.1952.sp004764 PMC1392413

[10] Hodgkin AL , Huxley AF. The dual effect of membrane potential on sodium conductance in the giant axon of loligo. J Physiol. 1952;116:497–506. 14946715 10.1113/jphysiol.1952.sp004719 PMC1392212

[11] Chanda B , Bezanilla F. Tracking voltage-dependent conformational changes in skeletal muscle sodium channel during activation. J Gen Physiol. 2002;120:629–645. 12407076 10.1085/jgp.20028679 PMC2229551

[12] Capes DL , Goldschen-Ohm MP, Arcisio-Miranda M, Bezanilla F, Chanda B. Domain IV voltage-sensor movement is both sufficient and rate limiting for fast inactivation in sodium channels. J Gen Physiol. 2013;142:101–112. 23858005 10.1085/jgp.201310998 PMC3727307

[13] West JW , Patton DE, Scheuer T, Wang Y, Goldin AL, Catterall WA. A cluster of hydrophobic amino acid residues required for fast Na^+^-channel inactivation. Proc Natl Acad Sci U S A. 1992;89:10910–10914. 1332060 10.1073/pnas.89.22.10910 PMC50452

[14] McPhee JC , Ragsdale DS, Scheuer T, Catterall WA. A critical role for the S4-S5 intracellular loop in domain IV of the sodium channel α-subunit in fast inactivation. J Biol Chem. 1998;273:1121–1129. 9422778 10.1074/jbc.273.2.1121

[15] Clairfeuille T , Cloake A, Infield DT, et al Structural basis of α-scorpion toxin action on Na_v_ channels. Science. 2019;363:eaav8573. 30733386 10.1126/science.aav8573

[16] Pan X , Li Z, Huang X, et al Molecular basis for pore blockade of human Na^+^ channel Na_v_1.2 by the μ-conotoxin KIIIA. Science. 2019;363:1309–1313. 30765605 10.1126/science.aaw2999

[17] Li X , Xu F, Xu H, et al Structural basis for modulation of human Na_v_1.3 by clinical drug and selective antagonist. Nat Commun. 2022;13:1286. 35277491 10.1038/s41467-022-28808-5 PMC8917200

[18] Jiang D , Shi H, Tonggu L, et al Structure of the cardiac sodium channel. Cell. 2020;180:122–134 e10. 31866066 10.1016/j.cell.2019.11.041 PMC6986426

[19] Jiang D , Banh R, Gamal El-Din TM, et al Open-state structure and pore gating mechanism of the cardiac sodium channel. Cell. 2021;184:5151–5162 e11. 34520724 10.1016/j.cell.2021.08.021 PMC8673466

[20] Pan X , Li Z, Zhou Q, et al Structure of the human voltage-gated sodium channel Na_v_1.4 in complex with β1. Science. 2018;362:eaau2486. 30190309 10.1126/science.aau2486

[21] Liu Y , Bassetto CAZ Jr, Pinto BI, Bezanilla F. A mechanistic reinterpretation of fast inactivation in voltage-gated Na^+^ channels. Nat Commun. 2023;14:5072. 37604801 10.1038/s41467-023-40514-4 PMC10442390

[22] Pan X , Li Z, Jin X, et al Comparative structural analysis of human Na_v_1.1 and Na_v_1.5 reveals mutational hotspots for sodium channelopathies. Proc Natl Acad Sci U S A. 2021;118:e2100066118. 33712547 10.1073/pnas.2100066118 PMC7980448

[23] Fan X , Huang J, Jin X, Yan N. Cryo-EM structure of human voltage-gated sodium channel Na_v_1.6. Proc Natl Acad Sci U S A. 2023;120:e2220578120. 36696443 10.1073/pnas.2220578120 PMC9945969

[24] Wolff M , Johannesen KM, Hedrich UB, et al Genetic and phenotypic heterogeneity suggest therapeutic implications in *SCN2A*-related disorders. Brain. 2017;140:1316–1336. 28379373 10.1093/brain/awx054

[25] Hieu NLT , Thu NTM, Ngan LTA, et al Genetic analysis using targeted exome sequencing of 53 Vietnamese children with developmental and epileptic encephalopathies. Am J Med Genet A. 2022;188:2048–2060. 35365919 10.1002/ajmg.a.62741

[26] Xu R , Thomas EA, Jenkins M, et al A childhood epilepsy mutation reveals a role for developmentally regulated splicing of a sodium channel. Mol Cell Neurosci. 2007;35:292–301. 17467289 10.1016/j.mcn.2007.03.003

[27] Berecki G , Howell KB, Heighway J, et al Functional correlates of clinical phenotype and severity in recurrent *SCN2A* variants. Commun Biol. 2022;5:515. 35637276 10.1038/s42003-022-03454-1 PMC9151917

[28] Berecki G , Howell KB, Deerasooriya YH, et al Dynamic action potential clamp predicts functional separation in mild familial and severe de novo forms of *SCN2A* epilepsy. Proc Natl Acad Sci U S A. 2018;115:E5516–E5525. 29844171 10.1073/pnas.1800077115 PMC6004444

[29] Berman HM , Westbrook J, Feng Z, et al The protein data bank. Nucleic Acids Res. 2000;28:235–242. 10592235 10.1093/nar/28.1.235 PMC102472

[30] Jo S , Kim T, Iyer VG, Im W. CHARMM-GUI: A web-based graphical user interface for CHARMM. J Comput Chem. 2008;29:1859–1865. 18351591 10.1002/jcc.20945

[31] Gotz AW , Williamson MJ, Xu D, Poole D, Le Grand S, Walker RC. Routine microsecond molecular dynamics simulations with AMBER on GPUs. 1. Generalized born. J Chem Theory Comput. 2012;8:1542–1555. 22582031 10.1021/ct200909j PMC3348677

[32] Salomon-Ferrer R , Gotz AW, Poole D, Le Grand S, Walker RC. Routine microsecond molecular dynamics simulations with AMBER on GPUs. 2. Explicit Solvent Particle Mesh Ewald. J Chem Theory Comput. 2013;9:3878–3888. 26592383 10.1021/ct400314y

[33] Tian C , Kasavajhala K, Belfon KAA, et al ff19SB: Amino-acid-specific protein backbone parameters trained against quantum mechanics energy surfaces in solution. J Chem Theory Comput. 2020;16:528–552. 31714766 10.1021/acs.jctc.9b00591 PMC13071887

[34] Case DA , Aktulga HM, Belfon K, et al, eds. 3.4. Lipids. Amber 2021: Reference manual. University of California Press; 2021:51–54. https://ambermd.org/doc12/Amber21.pdf

[35] Izadi S , Anandakrishnan R, Onufriev AV. Building water models: A different approach. J Phys Chem Lett. 2014;5:3863–3871. 25400877 10.1021/jz501780a PMC4226301

[36] Joung IS , Cheatham TE III. Determination of alkali and halide monovalent ion parameters for use in explicitly solvated biomolecular simulations. J Phys Chem B. 2008;112:9020–9041. 18593145 10.1021/jp8001614 PMC2652252

[37] Åqvist J , Wennerström P, Nervall M, Bjelic S, Brandsdal BO. Molecular dynamics simulations of water and biomolecules with a Monte Carlo constant pressure algorithm. Chem Phys Lett. 2004;384:288–294.

[38] Loncharich RJ , Brooks BR, Pastor RW. Langevin dynamics of peptides: The frictional dependence of isomerization rates of N-acetylalanyl-N'-methylamide. Biopolymers. 1992;32:523–535. 1515543 10.1002/bip.360320508

[39] Hopkins CW , Le Grand S, Walker RC, Roitberg AE. Long-Time-Step long-time-step molecular dynamics through hydrogen mass repartitioning. J Chem Theory Comput. 2015;11:1864–1874. 26574392 10.1021/ct5010406

[40] Ryckaert JP , Ciccotti G, Berendsen HJC. Numerical-integration of cartesian equations of motion of a system with constraints—molecular-dynamics of N-alkanes. J Comput Phys. 1977;23:327–341.

[41] Gowers R , Linke M, Barnoud J, et al MDAnalysis: A Python package for the rapid analysis of molecular dynamics simulations. In: *Proceedings of the 15th Python in Science Conference*. 2016. doi: 10.25080/Majora-629e541a-00e

[42] Bouysset C , Fiorucci S. ProLIF: A library to encode molecular interactions as fingerprints. J Cheminform. 2021;13:72. 34563256 10.1186/s13321-021-00548-6 PMC8466659

[43] Humphrey W , Dalke A, Schulten K. VMD: Visual molecular dynamics. J Mol Graph. 1996;14:33–38, 27–8. 8744570 10.1016/0263-7855(96)00018-5

[44] Suh D , Feng S, Lee H, et al CHARMM-GUI Enhanced sampler for various collective variables and enhanced sampling methods. Protein Sci. 2022;31:e4446. 36124940 10.1002/pro.4446 PMC9601830

[45] Bauer P , Hess B, Lindahl E. GROMACS 2022.5 Manual. Published online 12 July 2023. 10.5281/zenodo.8134388

[46] Huang J , Rauscher S, Nawrocki G, et al CHARMM36m: An improved force field for folded and intrinsically disordered proteins. Nat Methods. 2017;14:71–73. 27819658 10.1038/nmeth.4067 PMC5199616

[47] Jorgensen WL , Chandrasekhar J, Madura JD, Impey RW, Klein ML. Comparison of simple potential functions for simulating liquid water. J Chem Phys. 1983;79:926–935.

[48] Parrinello M , Rahman A. Polymorphic transitions in single crystals: A new molecular dynamics method. J Applied Phys. 1981;52:7182–7190.

[49] Evans DJ , Holian BL. The Nose–Hoover thermostat. J Chem Phys. 1985;83:4069–4074.

[50] Takacs DS , Houck KM, Seto E, Anderson A. Genetic etiologies of early-onset epileptic encephalopathy with burst suppression identified by whole exome sequencing. *Annual Meeting of the American Epilepsy Society*. 2018; Abstract (3.177).

[51] Stapley BJ , Doig AJ. Hydrogen bonding interactions between glutamine and asparagine in α-helical peptides. J Mol Biol. 1997;272:465–473. 9325104 10.1006/jmbi.1997.1262

[52] Rienecker KDA , Poston RG, Saha RN. Merits and limitations of studying neuronal depolarization-dependent processes using elevated external potassium. ASN Neuro. 2020;12:1759091420974807. 33256465 10.1177/1759091420974807 PMC7711227

[53] Reyes AD , Rubel EW, Spain WJ. Membrane properties underlying the firing of neurons in the avian cochlear nucleus. J Neurosci. 1994;14:5352–5364. 8083740 10.1523/JNEUROSCI.14-09-05352.1994 PMC6577066

[54] Sheets MF , Hanck DA. Voltage-dependent open-state inactivation of cardiac sodium channels: Gating current studies with anthopleurin-A toxin. J Gen Physiol. 1995;106:617–640. 8576700 10.1085/jgp.106.4.617 PMC2229277

[55] Fleidervish IA , Gutnick MJ. Kinetics of slow inactivation of persistent sodium current in layer V neurons of mouse neocortical slices. J Neurophysiol. 1996;76:2125–2130. 8890326 10.1152/jn.1996.76.3.2125

[56] Stuhmer W , Conti F, Suzuki H, et al Structural parts involved in activation and inactivation of the sodium channel. Nature. 1989;339:597–603. 2543931 10.1038/339597a0

[57] Armstrong CM , Bezanilla F, Rojas E. Destruction of sodium conductance inactivation in squid axons perfused with pronase. J Gen Physiol. 1973;62:375–391. 4755846 10.1085/jgp.62.4.375 PMC2226121

[58] Hanck DA , Sheets MF. Modification of inactivation in cardiac sodium channels: Ionic current studies with anthopleurin-A toxin. J Gen Physiol. 1995;106:601–616. 8576699 10.1085/jgp.106.4.601 PMC2229278

[59] Ben-Shalom R , Keeshen CM, Berrios KN, An JY, Sanders SJ, Bender KJ. Opposing effects on Na_v_1.2 function underlie differences between *SCN2A* variants observed in individuals with autism spectrum disorder or infantile seizures. Biol Psychiatry. 2017;82:224–232. 28256214 10.1016/j.biopsych.2017.01.009 PMC5796785

[60] Li M , Jancovski N, Jafar-Nejad P, et al Antisense oligonucleotide therapy reduces seizures and extends life span in an *SCN2A* gain-of-function epilepsy model. J Clin Invest. 2021;131:e152079. 34850743 10.1172/JCI152079 PMC8631599

[61] Ragsdale DS , McPhee JC, Scheuer T, Catterall WA. Molecular determinants of state-dependent block of Na^+^ channels by local anesthetics. Science. 1994;265:1724–1728. 8085162 10.1126/science.8085162

[62] Kuo CC . A common anticonvulsant binding site for phenytoin, carbamazepine, and lamotrigine in neuronal Na^+^ channels. Mol Pharmacol. 1998;54:712–721. 9765515

[63] Zeng Z , Hill-Yardin EL, Williams D, O'Brien T, Serelis A, French CR. Effect of phenytoin on sodium conductances in rat hippocampal CA1 pyramidal neurons. J Neurophysiol. 2016;116:1924–1936. 27489371 10.1152/jn.01060.2015 PMC5144711

[64] Boiteux C , Vorobyov I, French RJ, French C, Yarov-Yarovoy V, Allen TW. Local anesthetic and antiepileptic drug access and binding to a bacterial voltage-gated sodium channel. Proc Natl Acad Sci U S A. 2014;111:13057–13062. 25136136 10.1073/pnas.1408710111 PMC4246943

[65] Belardinelli L , Liu G, Smith-Maxwell C, et al A novel, potent, and selective inhibitor of cardiac late sodium current suppresses experimental arrhythmias. J Pharmacol Exp Ther. 2013;344:23–32. 23010360 10.1124/jpet.112.198887

[66] Kahlig KM , Hirakawa R, Liu L, George AL Jr, Belardinelli L, Rajamani S. Ranolazine reduces neuronal excitability by interacting with inactivated states of brain sodium channels. Mol Pharmacol. 2014;85:162–174. 24202911 10.1124/mol.113.088492

