# Supplementary material for: Nav1.2 channel mutations preventing fast inactivation lead to SCN2A encephalopathy
Source: Brain. 2024 Jun 28;148(1):212–26. doi: 10.1093/brain/awae213 (PMC11706276; doi:10.1093/brain/awae213)
Supplement: awae213_Supplementary_Data [file awae213_supplementary_data.zip › brain-2024-00535-File008.pdf]

SUPPLEMENTARY MATERIAL

De novo *SCN2A* mutations preventing Na_v1.2 channel fast inactivation lead to early-infantile developmental and epileptic encephalopathy

Géza Berecki†, Elaine Tao†, Katherine B. Howell, Rohini K. Coorg, Erik Andersen, Kris Kahlig, Markus Wolff, Ben Corry[#], Steven Petrou[#]

†,[#] These authors contributed equally to this work

Corresponding authors
Email: geza.berecki@florey.edu.au, ben.corry@anu.edu.au, steven.petrou@florey.edu.au

This file includes:

Figures S1 to S4
Table S1
Legends for Movies S1 and S2
SI References

Other supporting materials for this manuscript include the following:

Movies S1 and S2

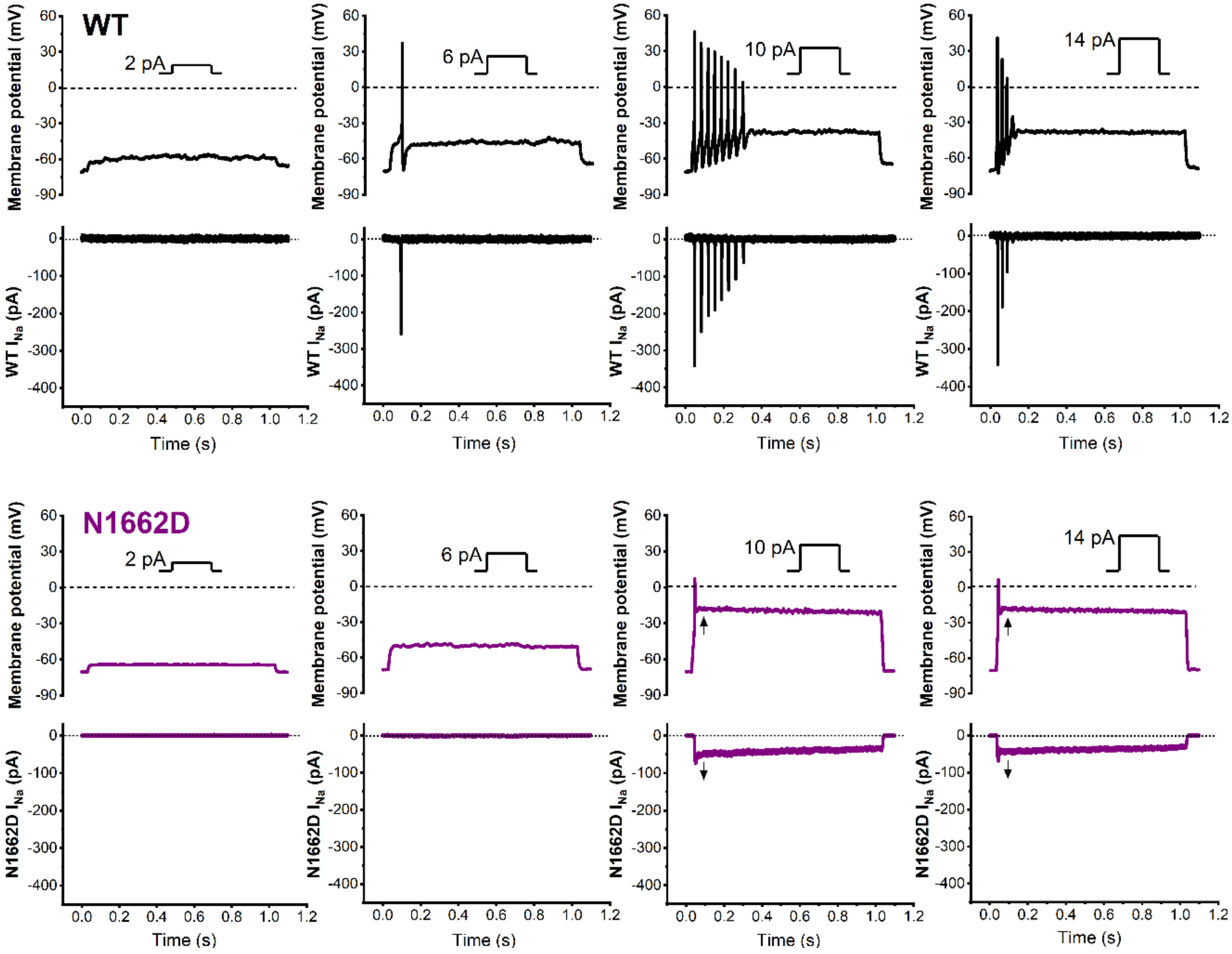

Figure S1. Membrane potential changes of hybrid neurons in the presence of heterologously expressed wild-type (WT) or N1662D Na_V1.2 sodium current (I_{Na}) in dynamic action potential clamp (DAPC) experiments. Hybrid neurons with WT I_{Na} show transient action

potential firing activity in response to depolarizing step current stimuli of increasing amplitude (insets) (top, black traces, from left to right); see the input-output relationships in Figure 5J). The implemented external WT I_{Na} traces, associated with action potential firing, are shown as downward deflections (bottom, black traces). Relative to WT, hybrid neurons with N1662D I_{Na} show an initial passive and small-magnitude depolarization in response to depolarizing step current stimuli (top, purple traces, first two panels from left), followed by a switch to a sustained depolarized state (arrows), in response to higher amplitude depolarizing stimuli (10 and 14 pA) (top, purple traces, last two panels on the right). The associated external N1662D I_{Na} traces show no channel activity (I_{Na} = 0) in hyperpolarized state (bottom, purple traces, first two panels from left) or result in sustained inward current due to non-inactivating N1662D channels. Dashed lines indicate the zero-mV membrane potential level; dotted lines indicate the zero-pA current level.

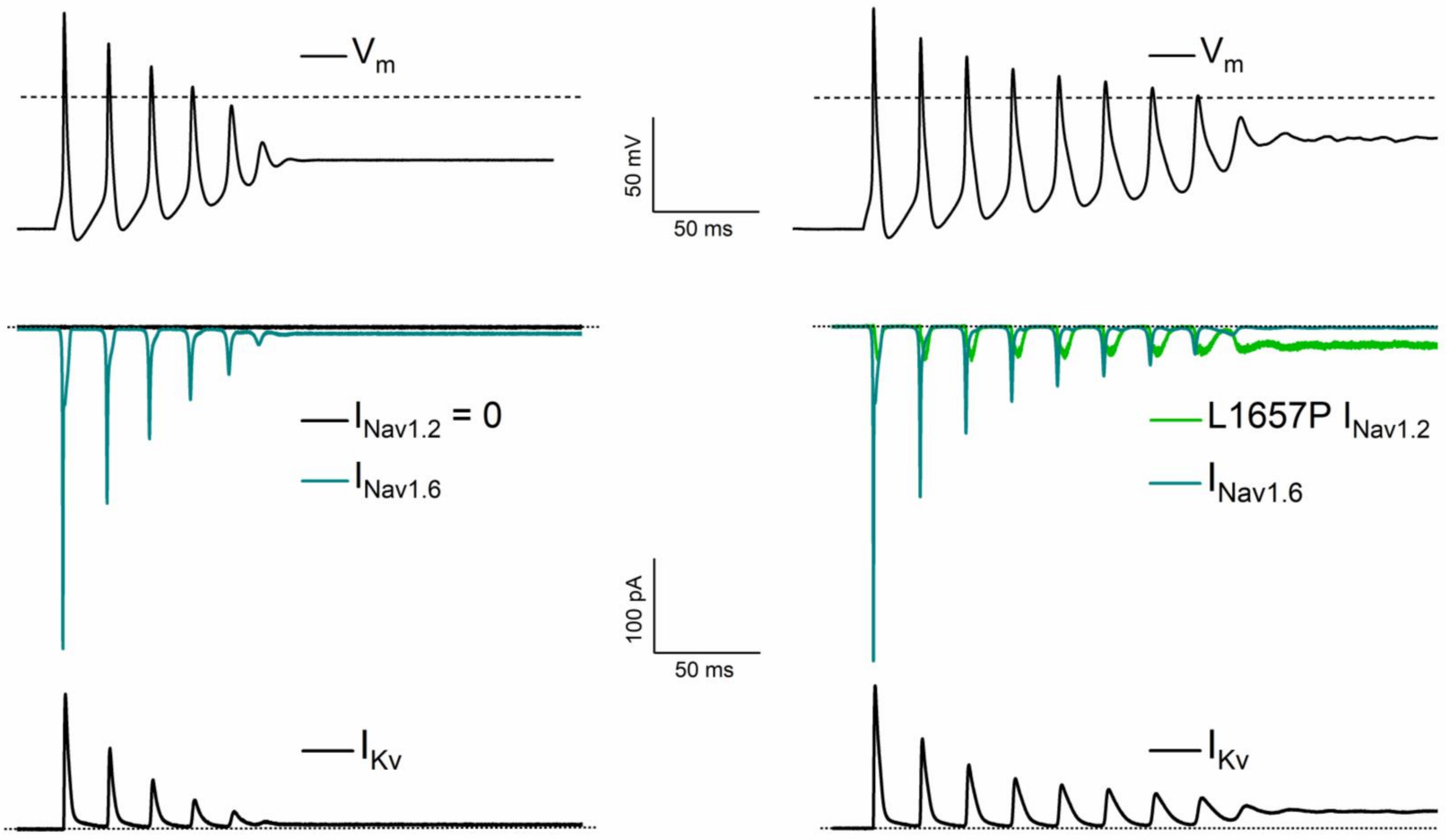

Figure S2. DAPC experiment demonstrating that a relatively small-amplitude external L16757P Na_v1.2 current ($I_{Nav1.2}$) increases action potential firing frequency in the presence of in silico Na_v1.6 current ($I_{Nav1.6}$). Action potential firing (top) in the absence (left) and presence (right) of L1657P $I_{Nav1.2}$. The action potential associated external L1657P $I_{Nav1.2}$ (green trace, middle right), in silico $I_{Nav1.6}$ (dark cyan traces, middle, left and right) and in silico potassium currents (IK_v, black traces, bottom, left and right) are shown. L1657P $I_{Nav1.2}$ was scaled to a peak amplitude corresponding to approximately 10 % of that of inward peak $I_{Nav1.6}$, resulting in a transient increase of the action potential firing transitioning into sustained depolarization of the membrane potential (V_m). Note that only the first 300 ms of the 1 s traces of V_m and current traces, elicited upon the injection of a 12-pA stimulus current, are shown.

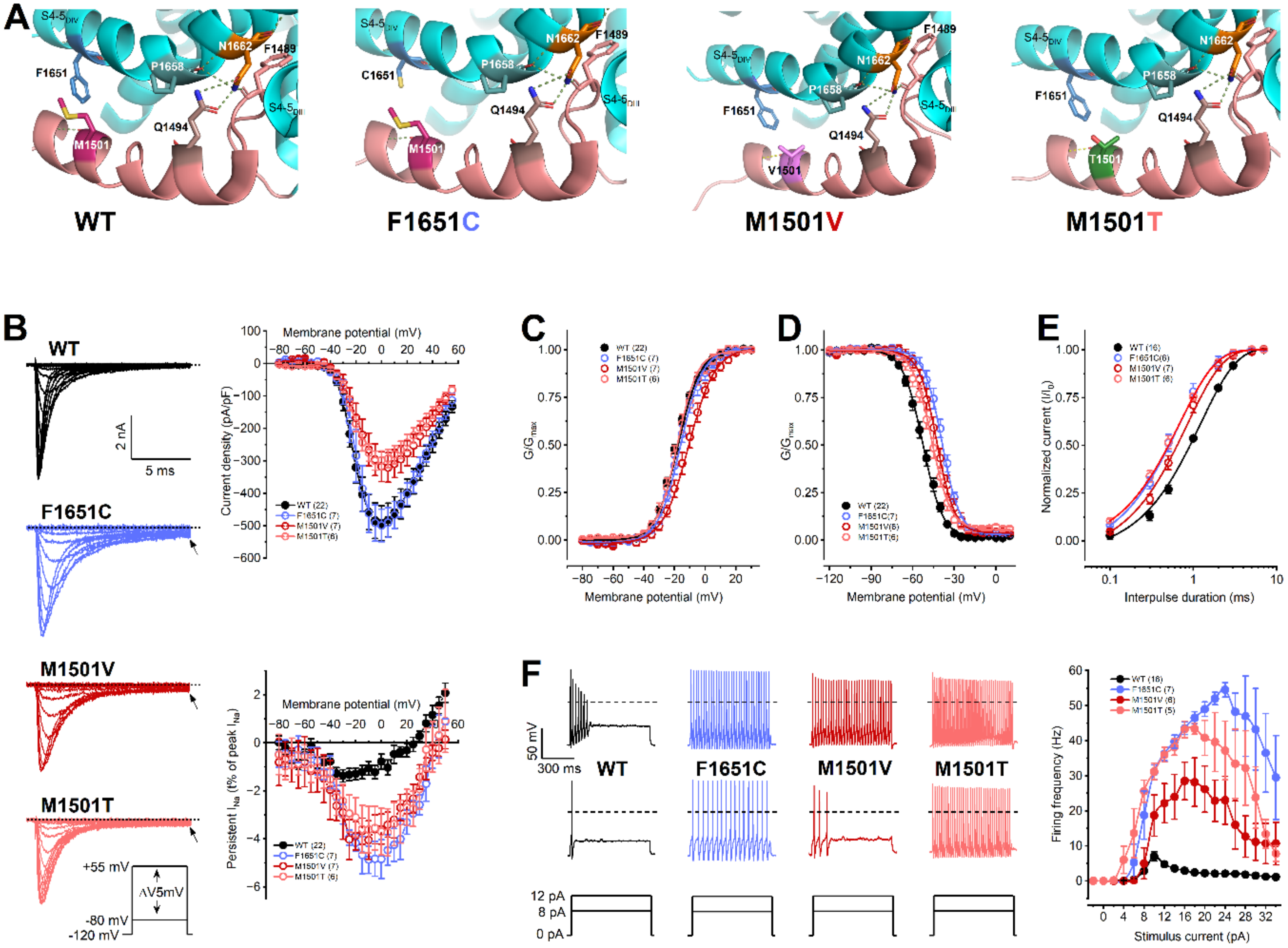

Figure S3. Biophysical properties and impact on hybrid neuron excitability of pathogenic F1651C, M1501V, and M1501T channels. **(A)** Zoomed-in views of the wild-type (WT) and mutant residues in segment 4-5 linker of domain IV (S4-5D_{IV}) or the inactivation gate (a-

helix in pink). Selected residues are represented as sticks, with the electronegative nitrogen amines (in blue), carbonyl oxygens (in red), hydroxyl group (red), and sulphur atom (yellow) of the amino acid side chains. Note the F1489 IFM motif residue in the inactivation gate. **(B)** Left: representative WT and mutant I_{Na} traces elicited in the voltage range between −80 and +10 mV. Top right: current density-voltage relationships. Top left: persistent inward sodium current (I_{Na})-voltage relationships (bottom); inset voltage protocol. Note the presence of persistent current with mutant channels (arrows) relative to WT. Dashed lines indicate zero current level. **(C)** Voltage dependence of activation **(D)** Voltage dependence of steady-state inactivation. **(E)** Recovery from fast inactivation. Data in C-E were fitted as described in Figure 1 (See Methods). See parameters of the fits and statistical evaluation in Table 1. **(F)** Representative action potential firing elicited by 8 and 12 pA step stimuli, and input-output relationships showing the effect of increasing stimulus strength on firing frequency in DAPC experiments. Dotted lines indicate zero membrane potential (V_m) level. Firing frequencies relative to WT were assessed using two-way ANOVA followed by Dunnett's post-hoc test (*$P < 0.05$). Data are presented as mean ± SEM; n, number of individual experiments in parentheses.

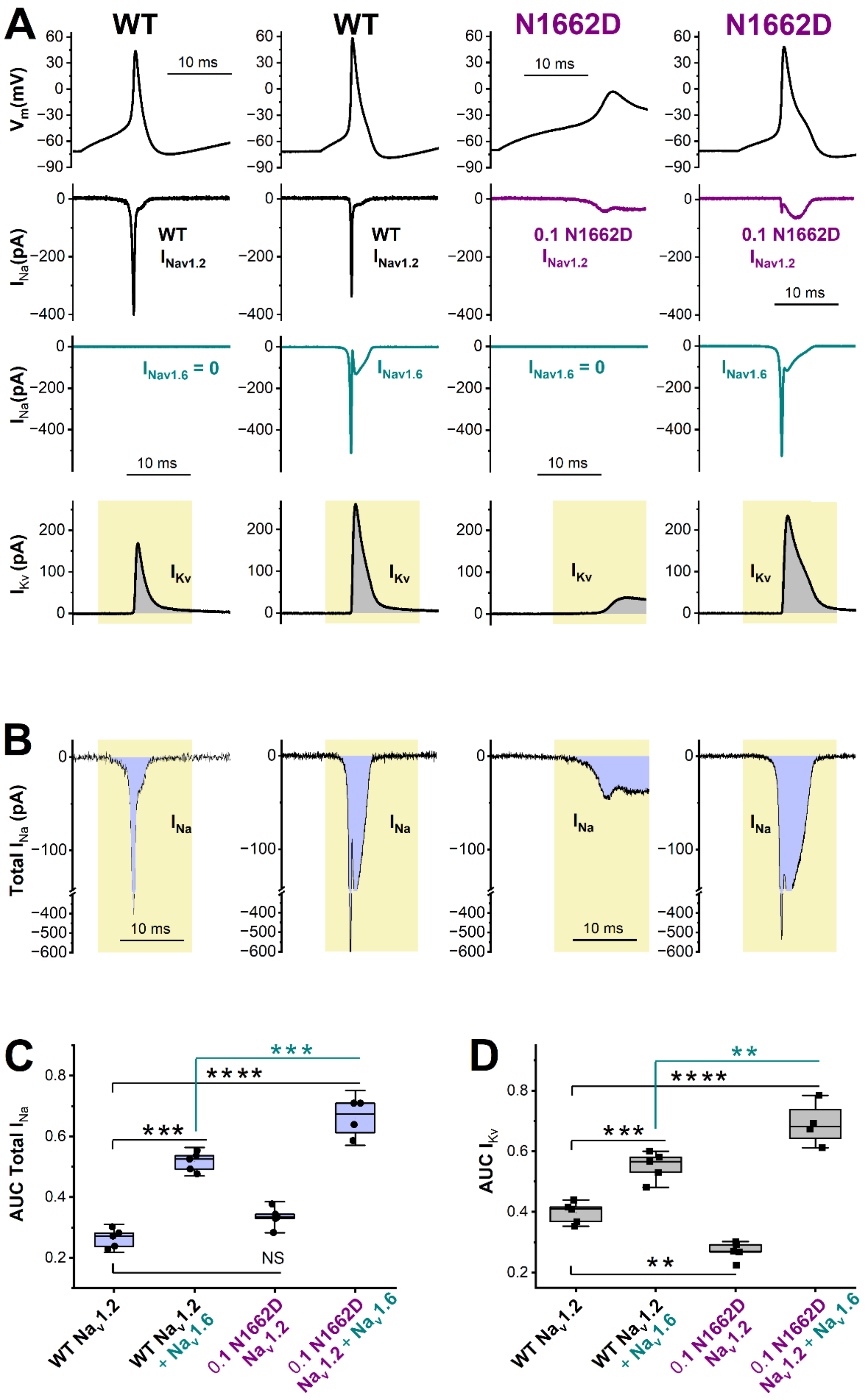

Figure S4. **Estimating the changes in sodium influx during single action potentials in DAPC experiments implementing wild-type (WT) or N1662D $Na_V1.2$ sodium current ($I_{Nav1.2}$). (A)** Time course of membrane

potential (V_m) changes (top row) and associated WT $I_{Nav1.2}$ or N1662D $I_{Nav1.2}$ (second row), virtual $Na_v1.6$ current ($I_{Nav1.6}$) (third row), and virtual potassium current (I_{Kv}) (bottom row). I_{Kv} was estimated by integrating the area under the outward current (AUC; shaded in dark gray) relative to the zero-current baseline during a 15-ms interval (yellow rectangular area) using OriginPro (Origin Lab Corporation, Northampton, MA, U.S.A). Both the WT $I_{Nav1.2}$ (n = 5) and N1662D $I_{Nav1.2}$ (n = 4) were tested in the presence or absence of virtual $I_{Nav1.6}$; 0.1 N1662D $Na_v1.2$ represents a 10-fold reduced peak current amplitude (~40 pA) relative to WT. Currents were not normalized to the size of axon initial compartment; in all experiments, the membrane capacitance of the axon initial compartment model corresponds to1.88 pF. **(B)** Total sodium current (I_{Na}), corresponding to WT $I_{Nav1.2} \pm I_{Nav1.6}$ or N1662D $I_{Nav1.2} \pm I_{Nav1.6}$ during action potentials. I_{Na} during the yellow-highlighted 15-ms interval, represented as the AUC (light gray). **(C** and **D)** Box and whisker plots of total sodium current (I_{Na}) and I_{Kv}, respectively. Shown are the individual AUC data (filled symbols), median, mean ± standard deviation, interquartile range (IQR), and whiskers (defined at 1.5 IQR). The AUC of total I_{Na} per spike was significantly greater in hybrid neurons incorporating 0.1 N1662D $I_{Nav1.2} + I_{Nav1.6}$ relative to WT $I_{Nav1.2} + I_{Nav1.6}$ or WT $I_{Nav1.2}$ alone. The AUC of the outward I_{Kv} per spike showed increases similar to those of total I_{Na} in hybrid neurons incorporating 0.1 N1662D $I_{Nav1.2} + I_{Nav1.6}$ relative to WT $I_{Nav1.2} + I_{Nav1.6}$ or WT $I_{Nav1.2}$ alone. This outward I_{Kv} increase is needed to counteract inward I_{Na} and facilitate the repolarization of the action potential. Conversely, the decreased I_{Kv} in hybrid neurons incorporating 0.1 N1662D $I_{Nav1.2}$ alone cannot initiate repolarization and leads to sustained depolarization in DAPC experiments. Asterisks indicate statistically significant differences using one-way ANOVA followed by Tukey's multiple comparison test; $^{**}P < 0.01$, $^{***}P < 0.001$, and $^{****}P < 0.0001$ compared with hybrid neurons incorporating WT $Na_v1.2$ alone; or $^{**}P < 0.01$ and $^{***}P < 0.001$ compared with WT $Na_v1.2 + Na_v1.6$.

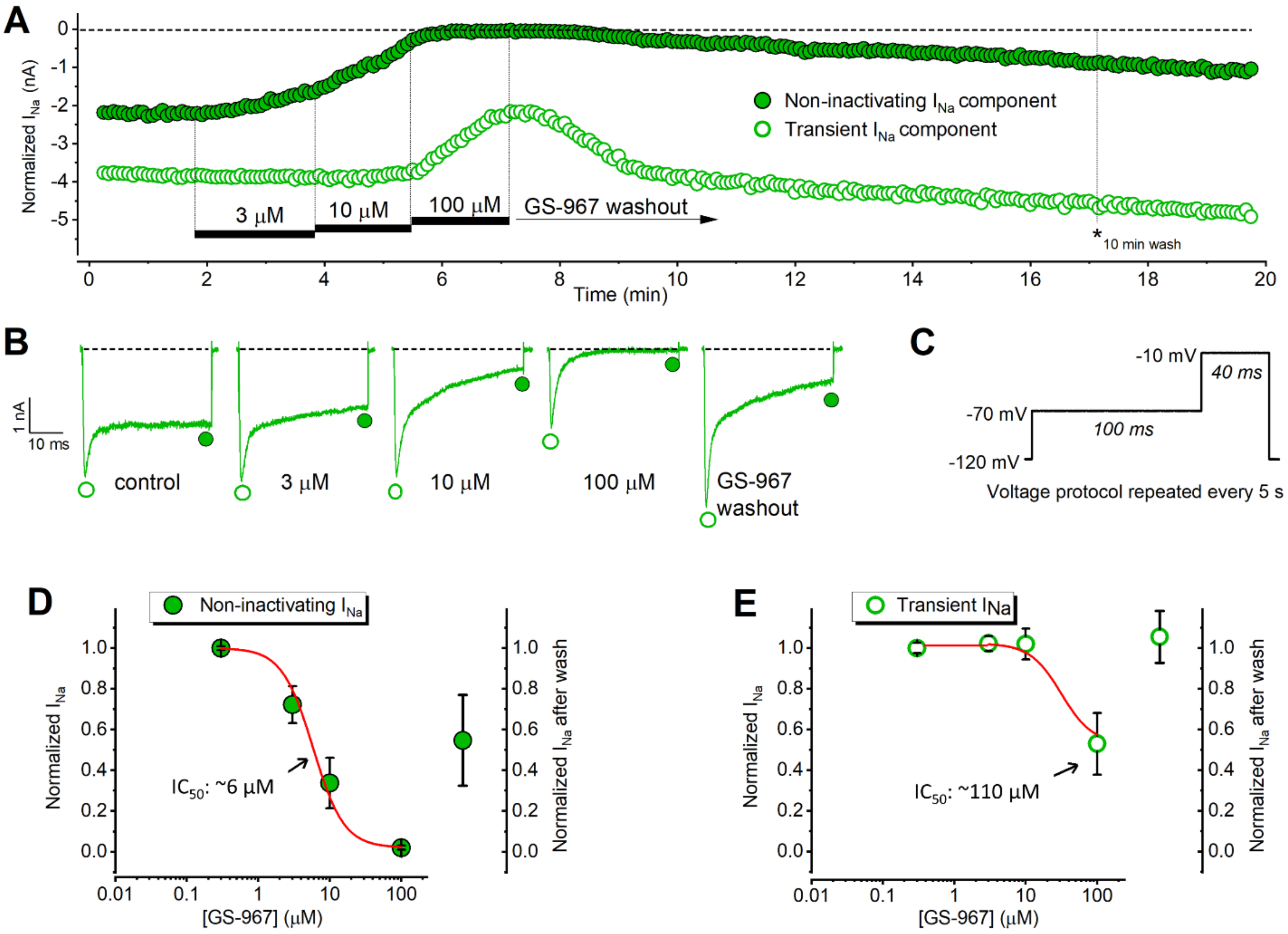

Figure S5. GS-967 inhibition of whole-cell L1657P Na$_V$1.2 currents in transiently transfected Chinese hamster ovary cells. (A) Representative experiment showing the effects of various concentrations of GS-967 and the recovery from GS-967 inhibition. The time

courses of the inward peak (hollow green circle) and non-inactivating current components (solid green circle), determined 40 ms after the onset of a –10 mV depolarizing step, is shown. **(B)** Representative current traces in the absence (control) and presence of GS-967, and 10 min after the onset of washout. **(C)** The voltage protocol used during the experiments. The GS-967 concentration-response of the non-inactivating **(D)** and transient **(E)** sodium current (I_{Na}) component, and I_{Na} recovery after 10 min washout. Data are mean ± SEM (n ≥ 3, for each data point). Data were analyzed off-line using Clampfit 9.2 (Molecular Devices) and Origin 2023 (Microcal Software Inc., Northampton, MA). Current amplitudes obtained in the presence of GS-967 (I) were normalized to current amplitudes obtained under control conditions (I_0). Concentration-response data were obtained by plotting averaged relative peak current amplitude (I/I_0) as function of GS-967 concentration ([GS-967]). The half-maximal inhibitory concentration (IC_{50}) values, shown in panels D and E, were determined after fitting the Hill equation $I = I_0\{[GS\text{-}967]^h/(IC_{50} + [GS\text{-}967]^h)\}$ to concentration-response data, where h is the Hill coefficient (slope). The percentage of inhibition was determined as $100 - I/I_0 \cdot 100$, whereas the I_{Na} fraction recovered from inhibition was defined as $[(Irec - I)/(I - I_0)]$, where I_{rec} is the current amplitude after 10 min washout.

Table S1. Site-directed mutagenesis primers

Primers	Sequences (5' to 3' direction)
N1662D-For	ATGTCCCTTCCTGCGTTGTTTGACATCGGCCTC
N1662D-Rev	GAGGCCGATGTCAAACAACGCAGGAAGGGACAT
Q1494A-For	GGTCAAGACATTTTTATGACAGAAGAAGCGAAGAAATACTACAATGCAATGAAAAA
Q1494A-Rev	TTTTTCATTGCATTGTAGTATTTCTTCGCTTCTTCTGTCATAAAAATGTCTTGACC
Q1494E-For	GTCAAGACATTTTTATGACAGAAGAAGAGAAGAAATACTACAATGCAATG
Q1494E-Rev	CATTGCATTGTAGTATTTCTTCTCTTCTTCTGTCATAAAAATGTCTTGAC
Q1494L-For	CAAGACATTTTTATGACAGAAGAACTGAAGAAATACTACAATGCAATGAAA
Q1494L-Rev	TTTCATTGCATTGTAGTATTTCTTCAGTTCTTCTGTCATAAAAATGTCTTG
Q1494K-For	GTCAAGACATTTTTATGACAGAAGAAAAGAAGAAATACTACAATGCAATG
Q1494K-Rev	CATTGCATTGTAGTATTTCTTCTTTTCTTCTGTCATAAAAATGTCTTGAC

Abbreviations: For, forward; Rev, reverse.

Movie S1 (separate file). Stability of the DIII-IV linker and IFM motif binding in wild-type (WT) $Na_v1.2$, captured in 1 µs of unbiased molecular dynamics simulation.

Movie S2 (separate file). Dissociation of the DIII-IV linker and IFM motif in the N1662D $Na_v1.2$ variant, captured in 1 µs of unbiased molecular dynamics simulation.
